# Association of mixed polycyclic aromatic hydrocarbons exposure with hearing loss and the mediating role of blood cell markers of inflammation in U.S. adults

**DOI:** 10.3389/fpubh.2024.1410601

**Published:** 2024-11-27

**Authors:** Peixuan Shen, Dan Hu, Meiyue Shen, Tingwei Du, Longzhu Zhao, Miaomiao Han, Ruihan Song, Rongrong Guo, Xiaochuan Lu, Shengnan Liu, Xiaoli Shen

**Affiliations:** ^1^Department of Epidemiology and Health Statistics, School of Public Health, Qingdao University, Qingdao, China; ^2^Licang District Center for Disease Control and Prevention, Qingdao, China

**Keywords:** hearing loss, polycyclic aromatic hydrocarbons, inflammation, NHANES, mixed exposure, Bayesian kernel machine regression (BKMR)

## Abstract

**Introduction:**

Studies on the effects of polycyclic aromatic hydrocarbons (PAHs) on hearing loss (HL) are limited and often focus on individual PAH compounds. The present study aimed to explore the individual and combined effects of PAH exposure on hearing loss, with a focus on the mediating role of inflammatory blood cell markers.

**Methods:**

This cross-sectional study included 1,409 participants from 3 cycles of the National Health and Nutrition Examination Survey (2001–2002, 2003–2004, and 2011–2012). Seven monohydroxylated PAH metabolites (OH-PAHs) in the urine were measured. Multivariable logistic regression, weighted quantile sum (WQS) regression, and Bayesian kernel machine regression (BKMR) were applied to assess both the individual and combined effects of OH-PAHs on hearing loss. Moreover, mediation analysis was employed to examine the mediating role of inflammatory blood cell markers in these associations.

**Results:**

Among the 1,409 participants, 59.1% had hearing loss. The WQS model revealed a positive association between PAH mixtures and HL (OR: 1.290; 95% CI: 1.042, 1.597), LFHL (OR: 1.363; 95% CI: 1.130, 1.644), and HFHL (OR: 1.299; 95% CI: 1.022, 1.626). Additionally, the WQS model identified hydroxynaphthalene (1-OHNAP) primarily contributed to HL and LFHL, while 2-hydroxyfluorene (2-OHFLU) was the primary contributor to HFHL. BKMR analysis demonstrated positive associations between PAH mixtures and all three types of hearing loss. Mediation analysis revealed that the association between OH-PAHs and LFHL was mediated by neutrophil (NEU) and basophil (BAS) counts.

**Discussion:**

These results confirmed that exposure to PAH mixtures was positively associated with the odds of hearing loss and that inflammatory blood cell markers mediated this association.

## 1 Introduction

Hearing loss (HL) is the fourth leading cause of disability worldwide (1), which affects approximately 5% of the global population (2). It poses a significant economic burden, with estimated costs reaching $19.4 billion annually in the United States (3). Beyond its economic impact, hearing loss reduces individuals' life quality and affects their physical and psychological wellbeing, leading to conditions such as dementia (4), depression (5), diabetes (6), and cardiovascular diseases (7). Factors including aging (8), genetic mutations (9), noise exposure (10), and environmental pollutants all contribute to the etiology of hearing loss (11).

Polycyclic aromatic hydrocarbons (PAHs) are widespread persistent organic pollutants. They are produced through the incomplete combustion of fossil fuels and released by wildfires and volcanic activity (12, 13). PAHs can be absorbed quickly into the body through inhalation, ingestion, or skin contact (14). Long-term exposure to PAH has been associated with an increased risk of cardiovascular diseases (14), lung function damage (15), cancer (16), and impaired cognitive performance (17). However, studies investigating the relationship between PAH exposure and hearing loss remain limited. A cross-sectional study conducted in the United States found that 3-Hydroxyfluorene and 2-Hydroxyfluorene were associated with an increased prevalence of hearing loss in adults and adolescents (18). Chou et al. reported elevated hearing thresholds in adults exposed to PAHs (19). Both studies employed multiple logistic regression models to assess the effects of individual PAH exposure on hearing loss. However, in real-world scenarios, people are often exposed to multiple PAHs simultaneously, which may have non-linear and non-additive effects on hearing loss. Hence, it is necessary to explore potential associations between PAH mixtures and hearing loss.

Several studies have indicated that PAHs can induce inflammatory reactions (20, 21). Chronic inflammation may lead to hearing loss by affecting the function of the blood barrier, signal transmission, and hair cell function (22, 23). White blood cells, as parts of the innate immune system and inflammatory cells in the inner ear, play a crucial role in detecting inflammation and overall human health (24). Liu et al. reported a positive association of PAH exposure with neutrophil and lymphocyte counts (25). Cao et al. reported that patients with severe hearing loss had higher neutrophil counts (26). However, the mediating role of inflammatory blood cell markers in the relationship between PAH and hearing loss has not yet been investigated.

In the present study, we aimed to explore the relationship between mixed PAH exposure and the odds of hearing loss by employing logistic regression, weighted quantile sum (WQS), and Bayesian kernel machine regression (BKMR) models. Additionally, we examined whether inflammatory blood cell markers mediate this relationship.

## 2 Materials and methods

### 2.1 Study participants

The National Health and Nutrition Examination Survey (NHANES) is an ongoing series of cross-sectional surveys conducted every 2 years by the Centers for Disease Control and Prevention (CDC). To ensure a representative sample of the civilian, non-institutionalized U.S. population, a multistage probability sampling design was utilized. Data were collected through home interviews and physical examinations at the mobile examination center (MEC). The study protocol was reviewed and approved by the Study Ethics Review Board of the National Center for Health Statistics and all participants signed informed consent. Institutional Review Board (IRB) approval was obtained before data collection. Further details about NHANES are available online (https://www.cdc.gov/nchs/nhanes.htm).

In the present analysis, a total of 2,328 participants aged ≥ 20 with urinary PAH metabolites data and hearing-related information were first enrolled from three NHANES cycles (2001–2002, 2003–2004, and 2011–2012). Among these participants, 919 were excluded for the following reasons: (1) 481 had missing data on covariates and white blood cell counts; (2) 99 provided unreliable responses, defined as showing a ≥10 dB difference between two tests at the 1 kHz frequency (27); (3) 361 reported ear disorders, excessive cerumen, or abnormal otoscopy. Ultimately, 1,409 participants were included in the final analysis. The flowchart of recruitment appears in Supplementary Figure S1.

### 2.2 Audiometric measurements and hearing loss

All hearing measurements were conducted in a special soundproof room at the MEC. The hearing measurement equipment included an audiometer (Interacoustics Model AD226) with standard headphones (TDH-49P) and insert earphones (Etymotic EarTone 3A). In addition, an environmental noise survey was implemented using a Quest 1800 sound level monitor to ensure test quality. Seven frequencies (500, 1,000, 2,000, 3,000, 4,000, 6,000, and 8,000 Hz) with intensities ranging from −10 to 120 were used to evaluate the participants' hearing thresholds in both ears. The NHANES website provides comprehensive information on the audiometry procedures and analysis techniques used (https://wwwn.cdc.gov/Nchs/Nhanes/2015-2016/AUX_I.htm#AUXROEXC).

According to the latest World Health Organization standards, hearing loss was defined as a pure-tone average ≥20 dB in either ear at speech frequencies (500, 1,000, 2,000, and 4,000 Hz) (27). Low-frequency hearing loss (LFHL) was defined as a pure-tone average ≥20 dB at 500, 1,000, and 2,000 Hz in either ear, as well as high-frequency hearing loss (HFHL) was defined as a pure-tone average ≥20 dB at 3,000, 4,000, 6,000, and 8,000 Hz in either ear (28).

### 2.3 Determination of urinary PAH metabolites

Urinary monohydroxylated PAH metabolites (OH-PAHs) have been proposed as biomarkers of internal PAH level (29). OH-PAHs in urine were measured by gas chromatography tandem mass spectrometry (GC–MS/MS).

The urinary OH-PAHs analyzed in this study comprised 1-hydroxynaphthalene (1-OHNAP), 2-hydroxynaphthalene (2-OHNAP), 3-hydroxyfluorene (3-OHFLU), 2-hydroxyfluorene (2-OHFLU), 1-hydroxyphenanthrene (1-OHPHE), 1-hydroxypyrene (1-OHPYR), and 2 & 3-hydroxyphenanthrene (2 & 3-OHPHE). Concentrations below the lower detection limit were replaced by the square root of the detection limit divided by 2. To account for impact of diluted urine, OH-PAHs concentrations were adjusted for urinary creatinine. The urinary OH-PAH concentration (ng/L) was divided by urinary creatinine (mg/dl) and multiplied by 0.01 (30), residual concentration expressed as nanograms of PAHs per gram of creatinine (ng/g Cr).

### 2.4 Measurement of inflammatory blood cell markers

Leukocyte counts were used to reflect systemic inflammation, including segmental neutrophil count (NEU), monocyte count (MON), lymphocyte count (LYM), basophil count (BAS), and eosinophilic count (EOS). During all cycles, blood samples were collected and processed in accordance with the NHANES Laboratory/Medical Technologists Procedures Manual (LPM). Whole blood cells were analyzed with a Coulter^®^ DxH800 analyzer, and blood cell distribution was determined for all participants.

### 2.5 Covariates

The covariates in this study included demographic information (age, sex, ethnicity, educational level, poverty-ratio index, body mass index and marital status), lifestyle information (smoking and drinking status), medical history (diabetes, hypertension and cardiovascular disease), noise exposure (occupational, firearm and recreational noise) and use of ototoxic medication. These covariates were selected based on their association with hearing loss and the findings of previous studies (6, 31–33). The categories for each covariate were as follows: Age was divided into three categories: 20–40, 41–60, and 61 years or older. Ethnicity was categorized as Mexican American, white (non-Hispanic), black (non-Hispanic), other Hispanic or other (including multiracial) race. Education level was divided into less than high school, high school, and more than high school. Marital status was grouped as married/cohabiting, widowed/divorced/separated, and never married. The poverty income ratio (PIR) was classified into two categories to measure family income, with a PIR < 1 being poverty. Body mass index (BMI, kg/m^2^), calculated as weight in kilograms divided by height in meters squared, was classified as underweight and normal weight (BMI < 25), overweight (25 ≤ BMI < 30), or obese (BMI ≥ 30) (34). Smoking status was classified as lifetime smoking of at least 100 cigarettes or not. Drinking status was categorized as consuming 12 or fewer alcoholic beverages per year or not. Diabetes was defined based on a fasting blood glucose level ≥ 126 mg/dL, a 2-h oral glucose tolerance test ≥ 200 mg/dL, a glycated hemoglobin level ≥ 6.5%, or a self-reported physician diagnosis or use of antihyperglycemic medication. Hypertension was defined as a mean systolic blood pressure ≥ 140 mmHg, diastolic blood pressure ≥ 90 mmHg, or self-reported physician diagnosis or use of antihypertensive medication. Cardiovascular disease (CVD) was defined as a self-reported diagnosis of congestive heart failure, coronary heart disease, angina, heart attack, or stroke. Noise exposure (occupational, firearm, and recreational) was self-reported (35). Use of ototoxic medication was defined by self-reported use of aminoglycoside, antineoplastic drugs, non-steroidal anti-inflammatory drugs, loop diuretics, macrolides, salicylates, and antimalarials within the past month (31, 36). The final form of our dataset with additional details could be found in the Supplementary Data sheet 2.

### 2.6 Statistical methods

The general characteristics of participants were presented as the means ± SDs for continuous variables with a normal distribution, and as medians with interquartile ranges (IQRs) for other variables. In addition, the frequency (percentage) was applied to express categorical variables. To assess the differences between each group with hearing loss and normal group, the chi-square test was employed for categorical variables. All OH-PAH concentrations were log-transformed to ensure normality. Pearson correlation coefficients were calculated to display the correlation among seven OH-PAHs.

First, the concentrations of urinary OH-PAHs were divided into quartiles. Weighted multivariable logistic regression was then fitted to estimate the associations between OH-PAHs levels and hearing loss (HL, LFHL and HFHL) with odds ratios (ORs) and 95% confidence intervals (CIs). Linear trends were tested by using the median value of each category of OH-PAH as a continuous variable in the models. Additionally, restricted cubic splines (RCS) analysis was employed to explore the dose-response association between ∑OH-PAHs and hearing loss (HL, LFHL, and HFHL).

Subsequently, the contribution of each OH-PAH and the effects of exposure to PAH mixtures on hearing loss were evaluated utilizing weighted quantile sum (WQS) regression analysis (37). The combined effects of exposure to seven OH-PAHs were represented by the WQS index. In addition, the WQS regression model assessed the contribution of each variable (OH-PAH) to the WQS index through a bootstrapping approach, where the weights of all components summed to 1. Data were randomly divided into validation (60%) and training (40%) datasets, with the bootstrap set to 10,000.

A Bayesian kernel machine regression (BKMR) model was further constructed. The new statistical model uses kernel functions to investigate both the individual and combined effects of mixtures on health (38). We applied Markov chain Monte Carlo (MCMC) algorithms to the models to examine the individual and combined effects of PAH mixtures on three types of hearing loss, using 1,008 random seeds and 1,000 iterations (39). The following results were derived by the BKMR model: (1) the combined effect of all OH-PAH mixtures at different percentiles on hearing loss compared with the 50th percentile; (2) the effect of a single OH-PAH exposure on hearing loss when other OH-PAHs were fixed at the 20th, 50th, or 70th percentile; (3) the presence of any interaction between two variables. A detailed description of the WQS regression and BKMR model can be found in a previous study (40).

Additionally, a linear regression model was fitted to explore the association between PAHs and inflammatory markers, and a binary logistic regression model was fitted to explore the association between inflammatory blood cell markers and hearing loss. Then, we conducted a mediation analysis to assess whether inflammatory cell markers mediated the effects of OH-PAH mixtures on hearing loss. The mediation effect model assumes the association between OH-PAH mixtures (X) and hearing loss (Y) is mediated by five white blood cell (WBC) counts (M) in this study (41). The total effect (TE) of PAHs on hearing loss can be divided into two parts: direct effect (DE) and indirect effect (IE) with the proportion of IE in TE calculated to determine the extent of inflammation mediated by these cell markers. All mediation analyses used 1,000 bootstrap samples to generate stable estimates and confidence intervals (42). In the sensitivity analyses, inverse probability weighting (IPW) was utilized in the multivariable logistic regression models to minimize potential selection bias. The propensity score (PS) was conducted to represent the predicted probability of participation. Then the inverse weight of each participant was calculated as 1/PS. This approach has been proven effective in avoiding potential selection bias in observational studies (43, 44). Additionally, the data from 03-04 and 11-12 were utilized to evaluate the robustness of the results.

R (version 4.0.2) and SPSS (version 25.0) software were applied for the statistical analyses, and the R package “ggplot2” was used to create the graphs. The model adjusted for all the covariates, such as age, sex, race, educational level, marital status, PIR, BMI, smoking status, drinking status, diabetes status, hypertension status, cardiovascular disease status, noise exposure (occupational, firearm, recreational noise) and use of ototoxic medication. A value of *p* ≤ 0.05 was considered to demonstrate statistical significance.

## 3 Results

### 3.1 Participant characteristics

Table 1 describes the characteristics of the 1,409 study participants and the prevalence of hearing loss. The prevalence of HL, LFHL, and HFHL was 59.1, 40.5, and 79.4%, respectively. Compared to the normal group, participants in the HL, HFHL, and LFHL groups tended to be older, overweight or obese, and have a history of CVD, diabetes and hypertension. In the HL and HFHL groups, significant differences in sex and race were observed. Furthermore, groups with hearing loss also differed significantly in terms of other general characteristics, such as age, education, marital status, BMI, smoking status, history of CVD, diabetes, hypertension and occupational noise exposure.

**Table 1:** Characteristics of the study population and prevalence of hearing loss by characteristic in the NHANES 2001–2002, 2003–2004, and 2011–2012.

**Characteristics**	**Normal hearing**	**HL**		**LFHL**		**HFHL**	
		**n (ratio, %)**	***p***	**n (ratio, %)**	***p***	**n (ratio, %)**	***p***
Total (*n* = 1,409)		833 (59.1)		570 (40.5)		1,119 (79.4)	
**Sex**							
Male (*n =* 658)	119	424 (64.4)	< 0.001^**^	266 (40.4)	0.984	535 (81.3)	0.367
Female (*n =* 751)	147	409 (54.5)		304 (40.5)		584 (77.8)	
**Age**							
20 ≤ Age ≤ 40 (*n =* 674)	226	252 (37.4)	< 0.001^**^	159 (23.6)	< 0.001^**^	430 (63.8)	< 0.001^**^
41 ≤ Age ≤ 60 (*n =* 532)	39	391 (73.5)		264 (49.6)		487 (91.5)	
Age ≥ 61 (*n =* 203)	1	190 (93.6)		147 (72.4)		202 (99.5)	
**Race**							
Mexican American (*n =* 209)	29	135 (64.6)	< 0.001^**^	87 (41.6)	0.375	178 (85.2)	< 0.001^**^
Other Hispanic (*n =* 104)	18	62 (59.6)		39 (37.5)		86 (82.7)	
White (non-hispanic) (*n =* 621)	108	384 (61.8)		261 (42.0)		500 (80.5)	
Black (non-hispanic) (*n =* 320)	58	184 (57.5)		131 (40.9)		256 (80.0)	
Other race (*n =* 155)	53	68 (43.9)		52 (33.5)		99 (63.9)	
**Educational level**							
Below high school (*n* = 261)	27	543 (61.3)	< 0.001^**^	132 (50.6)	< 0.001^**^	232 (88.9)	< 0.001^**^
High school (*n =* 300)	37	171 (76.3)		138 (46.0)		260 (86.7)	
Above high school (*n =* 848)	202	119 (39.8)		300 (35.4)		627 (73.9)	
**Marital status**							
Married/living with partner (*n =* 886)	145	572 (64.3)	< 0.001^**^	368 (41.5)	< 0.001^**^	730 (82.4)	< 0.001^**^
Widowed/divorced/separated (*n =* 224)	15	191 (21.5)		121 (54.0)		206 (92.0)	
Never married (*n =* 299)	106	121 (14.3)		81 (27.1)		183 (61.2)	
**PIR**							
PIR ≤ 1.00 (*n =* 1,120)	205	663 (59.2)	0.379	450 (40.2)	0.539	897 (80.1)	0.272
PIR > 1.00 (*n =* 289)	61	170 (58.8)		120 (41.5)		222 (76.8)	
**BMI, kg/m** ^2^							
Under and normal weight (*n =* 448)	118	235 (52.5)	< 0.001^**^	176 (39.3)	< 0.001^**^	320 (71.4)	< 0.001^**^
Over weight (*n =* 454)	77	268 (59.0)		168 (37.0)		369 (81.3)	
Obese (*n =* 507)	71	330 (65.1)		226 (44.6)		430 (84.8)	
**Smoking status**							
No (*n =* 791)	186	420 (53.1)	< 0.001^**^	280 (35.4)	< 0.001^**^	580 (85.6)	< 0.001^**^
Yes (*n =* 618)	80	413 (66.8)		290 (46.9)		529 (74.6)	
**Drinking status**							
No (*n =* 356)	61	235 (61.5	0.274	170 (27.6)	0.173	310 (26.0)	0.348
Yes (*n =* 1,053)	205	655 (73.6)		447 (72.4)		884 (74.0)	
**Diabetes**							
No (*n =* 1,224)	256	680 (51.0)	< 0.001^**^	456 (37.3)	< 0.001^**^	947 (77.4)	< 0.001^**^
Yes (*n =* 185)	10	153 (82.7)		114 (61.6)		172 (93.0)	
**Hypertension**							
No (*n =* 942)	480	480 (57.9)	< 0.001^**^	324 (34.4)	< 0.001^**^	694 (73.7)	< 0.001^**^
Yes (*n =* 467)	353	353 (75.6)		246 (52.7)		425 (91.0)	
**Cardiovascular disease**							
No (*n =* 1,332)	261	767 (57.6)	< 0.001^**^	521 (39.1)	< 0.001^**^	1047 (78.6)	0.004^*^
Yes (*n =* 77)	5	66 (85.7)		49 (63.6)		72 (93.5)	
**Firearm noise exposure**							
No (*n =* 1,067)	201	631 (59.1)	0.951	448 (42.0)	0.327	844 (79.1)	0.962
Yes (*n =* 342)	65	202 (59.1)		122 (35.7)		275 (80.4)	
**Recreational noise exposure**							
No (*n =* 1,172)	213	690 (58.9)	0.306	471 (40.2)	0.372	939 (80.1)	0.132
Yes (*n =* 237)	53	143 (60.3)		99 (41.8)		180 (75.9)	
**Occupational noise exposure**							
No (*n =* 923)	207	493 (53.4)	< 0.001^**^	353 (38.2)	< 0.001^**^	698 (75.6)	< 0.001^**^
Yes (*n =* 486)	59	340 (70.0)		217 (44.7)		421 (86.6)	
**Ototoxic medication use**							
No (*n =* 1,383)	260	818 (59.1)	0.637	559 (40.4)	0.756	1099 (79.5)	0.613
Yes (*n =* 26)	6	15 (57.7)		11 (42.3)		20 (76.9)	

Participants without hearing loss was classified into normal hearing. *P-*values were calculated using Chi-square test for categorical variables, when comparing the characteristics between hearing loss group and normal hearing group.

BMI, body mass index; HFHL, high-frequency hearing loss; HL, hearing loss; LFHL, low-frequency hearing loss; NHANES, National Health and Nutrition Examination Survey; PIR, poverty income ratio.

^*^p < 0.05; ^**^p < 0.01.

*N* = 1,409.

### 3.2 Distribution and correlations of urinary OH-PAHs levels

**Figure 1:**
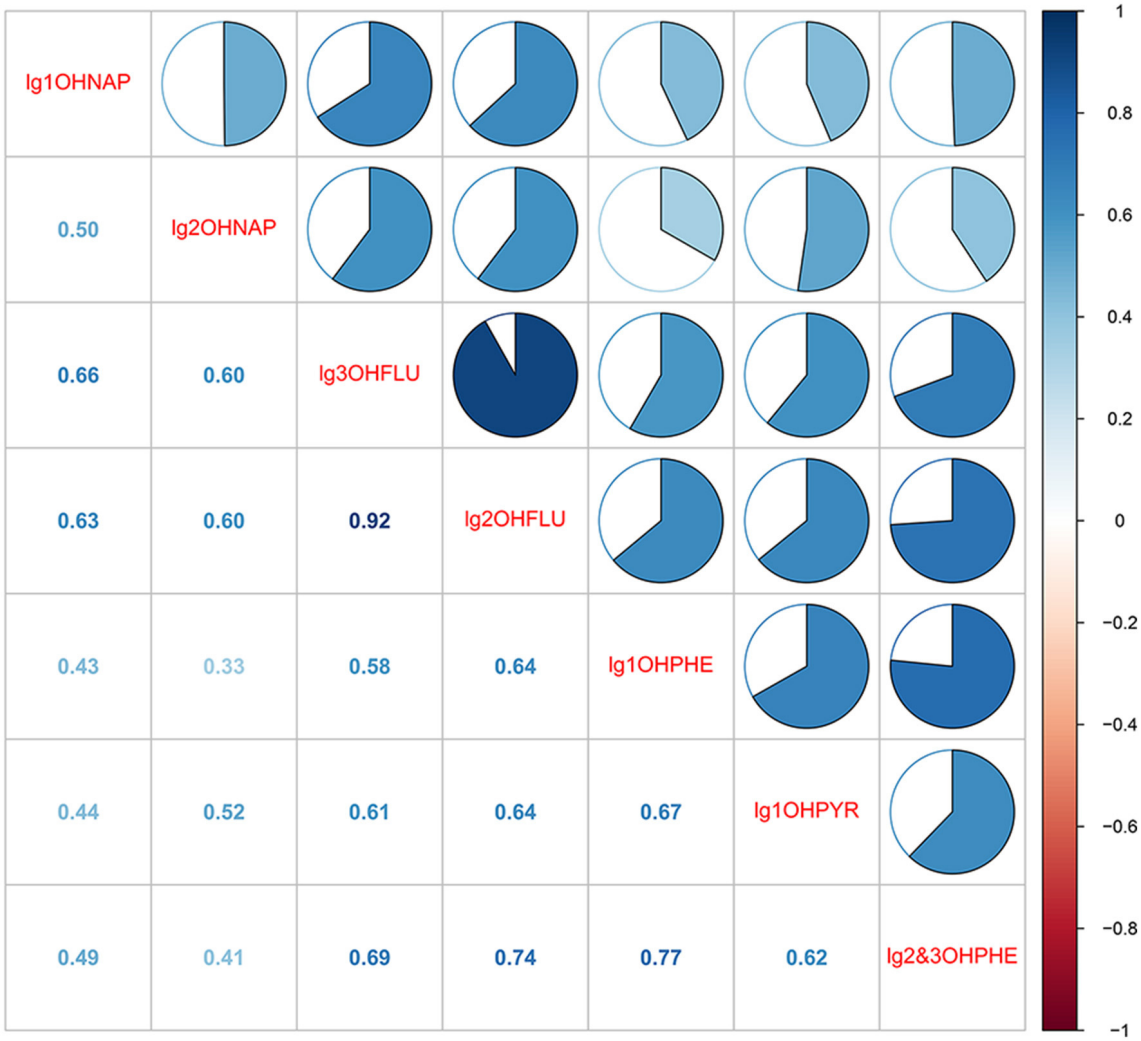
Pearson's correlations among the 7 OH-PAHs. All the correlations are statistically significant (*p* < 0.05). 1-OHNAP, 1-hydroxynaphthalene; 1-OHPHE, 1-hydroxyphenanthrene; 1-OHPYR, 1-hydroxypyrene; 2 & 3-OHPHE, 2 & 3-hydroxyphenanthrene; 2-OHFLU, 2-hydroxyfluorene; 2-OHNAP, 2-hydroxynaphthalene; 3-OHFLU, 3-hydroxyfluorene.

The detection rate and distribution of seven OH-PAHs in urine are presented in Supplementary Table S1. The detection rates of all the OH-PAHs were above 90%, with 2-OHNAP having the highest rate (94.2%) and concentration. The Pearson correlation coefficients (*r*) for the seven OH-PAHs are presented in Figure 1. Significant correlations among the concentrations of seven OH-PAHs were observed (*p* < 0.05), with correlation coefficients ranging from 0.33 to 0.92.

### 3.3 Multivariable logistic regression model

Table 2 presents the results of the multivariable logistic regression, adjusted for all covariates, to account for the effects of other OH-PAHs. The 2nd (OR: 1.802; 95% CI: 1.164–2.789) and 4th (OR: 2.091; 95% CI: 1.264–3.458) quantile of ∑OH-PAHs was positively associated with the odds of HL compared to the lowest quantile. And the 2nd (OR: 2.080; 95% CI: 1.247–3.468) and 4th (OR: 2.081; 95% CI: 1.322–3.274) quantile of ∑OH-PAHs were found to have a positive association with the odds of HFHL and LFHL, respectively. Moreover, the odds of HL and LFHL were significantly increased across quartiles of ∑OH-PAHs (*P* trend < 0.05). Conversely, the 2nd (OR: 0.602; 95% CI: 0.372, 0.934) and 4th (OR: 0.482; 95% CI: 0.261, 0.893) quantiles of 1-OHPYR were found to have a negative association with the odds of HL when compared with the lowest quantile. Compared with those in the lowest quantile, the 3rd quartile (OR: 2.278; 95% CI: 1.048, 4.950) of 2-OHFLU significantly increased the odds of HFHL. In contrast, the 4th (OR: 0.528; 95% CI: 0.294, 0.946) quantile of 2-OHNAP showed inverse associations with the odds of LFHL.

**Table 2:** Association between OH-PAHs exposure and hearing loss (*N* = 1,409), NHANES (2001–2002, 2003–2004, and 2011–2012).

**Urinary PAH metabolites, ng/g Cr**	**HL**		**HFHL**		**LFHL**	
	**OR (95% CI)**	***p***	**OR (95% CI)**	***p***	**OR (95% CI)**	***p***
**1-OHPYR**						
Continuous log-transformed	0.540 (0.287–1.014)	0.055	0.714 (0.334–1.527)	0.384	0.734 (0.417–1.289)	0.281
Quartile 1	Ref		Ref		Ref	
Quartile 2	0.602 (0.372–0.934)	0.039^*^	1.435 (0.803–2.565)	0.223	0.639 (0.398–1.025)	0.063
Quartile 3	0.724 (0.428–1.225)	0.228	0.941 (0.518–1.709)	0.841	0.891 (0.531–1.495)	0.662
Quartile 4	0.482 (0.261–0.893)	0.040^*^	0.700 (0.327–1.497)	0.357	0.967 (0.519–1.800)	0.914
*P* for trend	0.077		0.455		0.979	
**1-OHNAP**						
Continuous log-transformed	1.128 (0.788–1.614)	0.509	0.884 (0.654–1.510)	0.978	1.143 (0.806–1.621)	0.453
Quartile 1	Ref		Ref		Ref	
Quartile 2	0.901 (0.563–1.442)	0.664	0.781 (0.466–1.309)	0.348	1.224 (0.777–1.929)	0.383
Quartile 3	0.852 (0.521–1.392)	0.521	0.673 (0.386–1.174)	0.163	1.050 (0.635–1.734)	0.851
Quartile 4	1.108 (0.588–2.090)	0.751	1.336 (0.621–2.876)	0.459	1.348 (0.757–2.400)	0.310
*P* for trend	0.784		0.709		0.366	
**2-OHNAP**						
Continuous log-transformed	0.830 (0.506–1.359)	0.458	0.857 (0.484–1.519)	0.597	0.606 (0.382–0.961)	0.033^*^
Quartile 1	Ref		Ref		Ref	
Quartile 2	0.686 (0.430–1.095)	0.114	1.030 (0.613–1.731)	0.911	0.902 (0.571–1.424)	0.657
Quartile 3	0.789 (0.476–1.308)	0.358	0.799 (0.447–1.429)	0.449	0.652 (0.393–1.081)	0.097
Quartile 4	0.713 (0.379–1.339)	0.292	0.824 (0.422–1.610)	0.571	0.528 (0.294–0.946)	0.032^*^
*P* for trend	0.449		0.648		0.018^*^	
**1-OHPHE**						
Continuous log-transformed	1.602 (0.667–3.847)	0.292	1.420 (0.488–4.131)	0.520	1.118 (0.495–2.523)	0.789
Quartile 1	Ref		Ref		Ref	
Quartile 2	1.346 (0.843–2.150)	0.213	1.160 (0.686–1.960)	0.579	1.619 (0.990–2.648)	0.055
Quartile 3	1.461 (0.838–2.549)	0.181	1.500 (0.806–2.790)	0.200	1.461 (0.845–2.526)	0.175
Quartile 4	1.684 (0.837–3.385)	0.144	1.316 (0.607–2.852)	0.487	1.165 (0.594–2.286)	0.657
*P* for trend	0.169		0.424		0.886	
**2 & 3-OHPHE**						
Continuous log-transformed	0.634 (0.295–1.361)	0.242	0.421 (0.170–1.048)	0.063	0.993 (0.492–2.002)	0.984
Quartile 1	Ref		Ref		Ref	
Quartile 2	1.096 (0.664–1.808)	0.721	1.219 (0.696–2.131)	0.489	0.777 (0.466–1.298)	0.335
Quartile 3	0.929 (0.505–1.711)	0.813	0.929 (0.463–1.864)	0.835	0.855 (0.472–1.548)	0.605
Quartile 4	0.979 (0.444–2.161)	0.958	1.101 (0.440–2.756)	0.838	1.357 (0.658–2.801)	0.408
*P* for trend	0.717		0.970		0.401	
**3-OHFLU**						
Continuous log-transformed	1.145 (−0.463 to 2.833)	0.769	0.856 (0.292–2.504)	0.776	0.833 (0.351–1.976)	0.678
Quartile 1	Ref		Ref		Ref	
Quartile 2	0.971 (0.574–1.641)	0.912	1.083 (0.589–1.992)	0.797	0.900 (0.528–1.533)	0.698
Quartile 3	1.264 (0.619–2.582)	0.519	0.794 (0.366–1.721)	0.559	0.797 (0.401–1.585)	0.517
Quartile 4	1.990 (0.659–6.010)	0.222	0.611 (0.160–2.333)	0.471	0.820 (0.303–2.217)	0.696
*P* for trend	0.225		0.457		0.822	
**2-OHFLU**						
Continuous log-transformed	3.151 (0.990–10.025)	0.052	4.346 (1.008–18.746)	0.049^*^	3.942 (1.302–11.937)	0.015^*^
Quartile 1	Ref		Ref		Ref	
Quartile 2	1.056 (0.615–1.213)	0.844	1.149 (0.614–1.910)	0.649	1.071 (0.634–1.810)	0.896
Quartile 3	1.085 (0.517–2.278)	0.828	2.278 (1.048–4.950)	0.037^*^	1.353 (0.699–2.617)	0.481
Quartile 4	1.344 (0.449–4.027)	0.597	2.629 (0.654–10.568)	0.173	2.419 (0.966–6.062)	0.056
*P* for trend	0.464		0.244		0.042^*^	
Σ**OH-PAHs**						
Continuous log-transformed	1.646 (1.100–2.461)	0.015^*^	1.443 (1.011–2.059)	0.043^*^	1.544 (1.168–2.042)	0.002^**^
Quartile 1	Ref		Ref		Ref	
Quartile 2	1.802 (1.164, 2.789)	0.008^**^	2.080 (1.247–3.468)	0.005^**^	1.556 (0.990–2.445)	0.055
Quartile 3	1.293 (0.810, 2.064)	0.281	1.245 (0.750–2.067)	0.396	1.412 (0.734 1.775)	0.556
Quartile 4	2.091 (1.264, 3.458)	0.004^**^	1.597 (0.894–2.852)	0.114	2.081 (1.322–3.274)	0.002^**^
*P* for trend	0.019^*^		0.308		0.006^*^	

The model was adjusted for age, sex, race/ethnicity, education level, marital status, PIR, BMI, drinking, smoking, diabetes, hypertension, cardiovascular disease, occupational, firearm, recreational noise, and use of ototoxic medication.1-OHNAP, 1-hydroxynaphthalene; 1-OHPHE, 1-hydroxyphenanthrene; 1-OHPYR, 1-hydroxypyrene; 2 & 3-OHPHE, 2 & 3-hydroxyphenanthrene; 2-OHFLU, 2-hydroxyfluorene; 2-OHNAP, 2-hydroxynaphthalene; 3-OHFLU, 3-hydroxyfluorene; ∑OH-PAHs, total of PAH metabolites; *CI*, confidence interval; HFHL, high-frequency hearing loss; HL, hearing loss; LFHL, low-frequency hearing loss; *OR*, odds ratios; PAHs, polycyclic aromatic hydrocarbon.

*P* for trend across quartiles of urinary PAH metabolites was tested by including the median of each quartile of urinary PAH metabolites as a continuous variable in models.

^*^p < 0.05, ^**^p < 0.01.

When OH-PAHs were assessed as a continuous variable, ∑OH-PAHs were also positively associated with HL (OR: 1.646; 95% CI: 1.100–2.461), HFHL (OR: 1.443; 95% CI: 1.011–2.059), and LFHL (OR: 1.544; 95% CI: 1.168–2.042). Additionally, 2-OHFLU was positively associated with LFHL (OR: 3.942; 95% CI: 1.302–11.937) and HFHL (OR: 4.346; 95% CI: 1.008–18.746). In contrast, 2-OHNAP (OR: 0.606; 95% CI: 0.382–0.961) showed inverse associations with the odds of LFHL. RCS analysis indicated that log-transformed ∑OH-PAHs significantly increased the odds of HL, HFHL and LFHL (*p* for overall < 0.05) (Supplementary Figure S2). Besides the linear relationship in LFHL (*p* for non-linear = 0.174), ∑OH-PAHs demonstrated non-linear relationships with HL (*p* for nonlinear = 0.018) and HFHL (*p* for nonlinear = 0.011), with the cut-off for LFHL, HL, and HFHL were 0.651 (∑OH-PAHs concentration: 4.477 ng/g Cr), 0.661 (∑OH-PAHs concentration: 4.581 ng/g Cr), and 0.682 (∑OH-PAHs concentration: 4.842 ng/g Cr), respectively (Supplementary Figure S2).

### 3.4 WQS regression model

**Figure 2:**
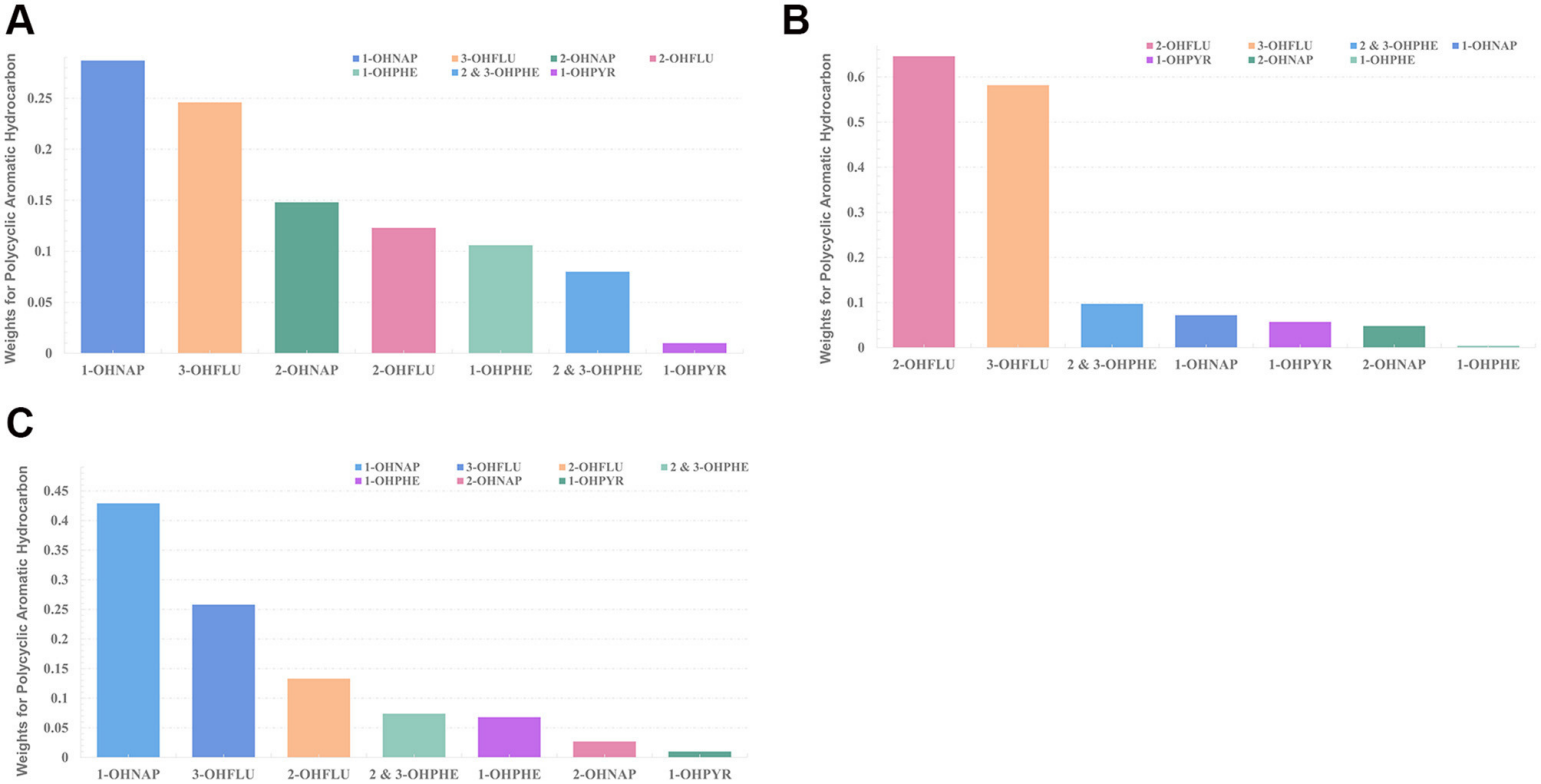
Weights of OH-PAHs from the WQS model for hearing loss [**(A)** HL; **(B)** HFHL; **(C)** LFHL]. The models were adjusted for age, sex, race/ethnicity, education level, marital status, PIR, BMI, drinking, smoking, diabetes, hypertension, cardiovascular disease, occupational, firearm, recreational noise, and use of ototoxic medication. 1-OHNAP, 1-hydroxynaphthalene; 1-OHPHE, 1-hydroxyphenanthrene; 1-OHPYR, 1-hydroxypyrene; 2 & 3-OHPHE, 2 & 3-hydroxyphenanthrene; 2-OHFLU, 2-hydroxyfluorene; 2-OHNAP, 2-hydroxynaphthalene; 3-OHFLU, 3-hydroxyfluorene; HFHL, high-frequency hearing loss; HL, hearing loss; LFHL, low-frequency hearing loss.

WQS regression model in both the positive and negative directions was implemented to evaluate the combined effects of PAH mixtures on hearing loss. As displayed in Table 3, after adjusting for all the covariates, the WQS indices of the OH-PAH mixtures were significantly positively associated with HL (OR: 1.290; 95% CI: 1.042–1.597), HFHL (OR: 1.299; 95% CI: 1.022–1.626), and LFHL (OR: 1.363; 95% CI: 1.130–1.644). Regarding the positive effect of OH-PAH mixtures on HL and LFHL, 1-OHNAP had the highest contribution to the WQS index (HL: weighted 0.287; LFHL: weighted 0.429) (Figures 2A, C; Supplementary Table S2). In the positive association between OH-PAH mixtures and HFHL, 2-OHFLU contributed the most to the WQS index (weighted 0.646) (Figure 2B; Supplementary Table S2).

**Table 3:** Association between the WQS index and hearing loss (*N* = 1,409), NHANES (2001–2002, 2003–2004, and 2011–2012).

	**OR**	**95% CI of OR**	***p***
HL	1.290	1.042–1.597	0.019^*^
HFHL	1.299	1.022–1.626	0.032^*^
LFHL	1.363	1.130–1.644	0.001^*^

The WQS model was adjusted for age, sex, race/ethnicity, education level, marital status, PIR, BMI, drinking, smoking, diabetes, hypertension, cardiovascular disease, occupational, firearm, recreational noise, and use of ototoxic medication. OR estimates represent the odds ratios of hearing loss when the WQS index of PAH mixtures was increased by 1 tertile.

CI, confidence interval; HFHL, high-frequency hearing; HL, hearing loss; LFHL, low-frequency hearing loss; OR, odds ratios.

^*^p < 0.05.

### 3.5 BKMR model

**Figure 3:**
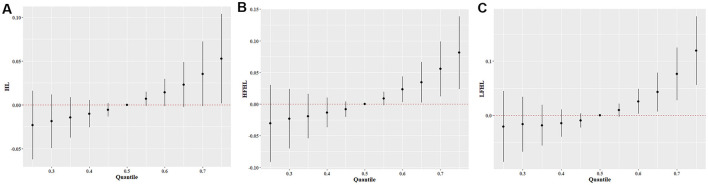
The combined effects of OH-PAH mixtures on HL **(A)**, HFHL **(B)**, and LFHL **(C)** in BKMR models. All models were adjusted age, sex, race/ethnicity, education level, marital status, PIR, BMI, drinking, smoking, diabetes, hypertension, cardiovascular disease, occupational, firearm, recreational noise, and use of ototoxic medication. HFHL, high-frequency hearing loss; HL, hearing loss; LFHL, low-frequency hearing loss.

The BKMR model was applied to analyze the individual and combined effects of urinary OH-PAH exposure on the three types of hearing loss. The overall associations between mixed OH-PAH exposure and hearing loss are presented in Figure 3. When all OH-PAHs were at or above the 75th percentile, a significantly positive association was observed between OH-PAH mixtures and HL compared to their median levels (Figure 3A). When all OH-PAHs were at or above the 60th percentile, a significantly positive association was found between OH-PAH mixtures and HFHL compared to their median levels (Figure 3B). Furthermore, when the OH-PAH concentration was at or above the 60th percentile, there was a significantly positive association between the OH-PAH mixtures and LFHL compared to the association between the OH-PAH concentration and the median level (Figure 3C).

**Figure 4:**
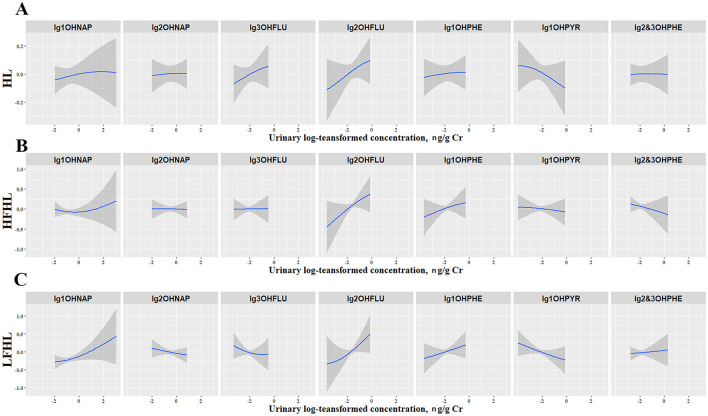
The univariate exposure–response functions and 95% confidence intervals for each OH-PAH with HL **(A)**, HFHL **(B)**, and LFHL **(C)** when fixing other chemicals at their 50th percentile. The shaded areas represent 95% confidence intervals. All models were adjusted for age, sex, race/ethnicity, education level, marital status, PIR, BMI, drinking, smoking, diabetes, hypertension, cardiovascular disease, occupational, firearm, recreational noise, and use of ototoxic medication. 1-OHNAP, 1-hydroxynaphthalene; 1-OHPHE, 1-hydroxyphenanthrene; 1-OHPYR, 1-hydroxypyrene; 2 & 3-OHPHE, 2 & 3-hydroxyphenanthrene; 2-OHFLU, 2-hydroxyfluorene; 2-OHNAP, 2-hydroxynaphthalene; 3-OHFLU, 3-hydroxyfluorene; HFHL, high-frequency hearing loss; HL, hearing loss; LFHL, low-frequency hearing loss.

Subsequently, the univariate exposure-response functions for seven OH-PAHs were assessed. When all other OH-PHAs were fixed at the 50th percentile, 1-OHNAP, 2-OHFLU, and 1-OHPHE were positively associated with the odds of all three types of hearing loss, while 1-OHPYR showed inverse associations with them (Figure 4). In addition, we examined the potential interactions among the seven OH-PAHs (Supplementary Figure S3). The interactions among the exposed variables were not significant, and the correlation curves were basically parallel.

### 3.6 Mediation analysis

First, linear regression analysis was conducted to explore the association between PAHs and inflammatory markers. After adjusting for all the covariates, OH-PAHs showed positive associations with NEU (β: 0.034; 95% CI: 0.021, 0.047), BAS (β: 0.050; 95% CI: 0.014, 0.790), LYM (β: 0.088; 95% CI: 0.024, 0.088) and NOM (β: 0.054; 95% CI: 0.011, 0.263) (Supplementary Figure S4). Second, a binary logistic regression model was fitted to explore the association between inflammatory blood cell markers and hearing loss. NEU was found positively associated with HL (OR: 1.090; 95% CI: 1.011, 1.176) and LFHL (OR: 1.125; 95% CI: 1.048, 1.208) (Supplementary Table S3). Similarly, BAS was positively associated with HL (OR: 8.324; 95% CI: 1.103, 62.813) and LFHL (OR: 19.927; 95% CI: 3.030, 131.639) (Supplementary Table S3).

**Figure 5:**
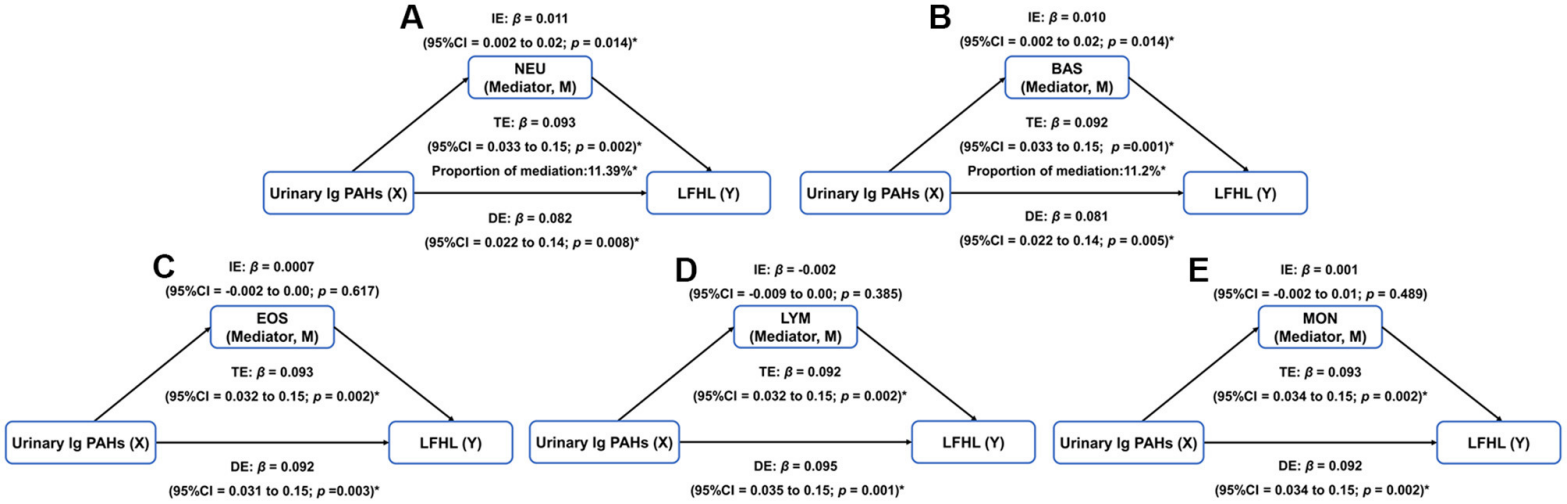
Mediation analysis of blood cell markers of inflammation on the association between PAH mixtures and LFHL. **(A)** NEU. **(B)** BAS. **(C)** EOS. **(D)** LYM. **(E)** MON. BAS, basophil count; DE, effect; EOS, eosinophilic count; IE, indirect effect; LFHL, low-frequency hearing loss; LYM, lymphocyte count; MON, monocyte count; NEU, neutrophil count; PAH, polycyclic aromatic hydrocarbon; TE, total effect. *p < 0.05.

Based on the above results, mediation analysis was performed to investigate whether the association between OH-PAHs and hearing loss was mediated by inflammatory blood cell markers (Figure 5; Supplementary Figures S5, S6). Results showed that both NEU and BAS significantly mediated the association between mixtures of PAHs and LFHL. As shown in Figures 5A, B, NEU accounted for 11.39% of mediation in the association between PAHs and LFHL was 11.39% (IE: β = 0.011; 95% CI: 0.002, 0.02; DE: β = 0.082; 95% CI: 0.022, 0.14; TE: β = 0.093; 95% CI: 0.033, 0.15), while BAS accounted for 11.2 % (IE: β = 0.010; 95% CI: 0.002, 0.02; DE: β = 0.081; 95% CI: 0.022, 0.14; TE: β = 0.092; 95% CI: 0.033, 0.15). In the sensitive analysis, inverse probability weighted analysis was conducted to minimize potential selection bias. The results were consistent with our main analysis and presented in Supplementary Table S4. Additionally, when the data from 03-04 and 11-12 cycles were conducted, the results were consistent with our results in the BKMR models (Supplementary Figures S7–S9). In the WQS models, PAH mixtures increased the odds of HL and LFHL, while no significant association between PAH mixtures and HFHL was observed (Supplementary Table S5).

## 4 Discussion

To our knowledge, this is the first study to assess the combined effects of PAH mixtures on hearing loss in U.S. adults by using three different statistical approaches. First, we found that PAH mixtures increased the odds of all three types of hearing loss. Second, the WQS model identified the primary contributions of 1-OHNAP to the positive associations between PAH mixtures and HL and LFHL, while 2-OHFLU was the primary contributor to the positive association between PAH mixtures and HFHL. Finally, mediation analysis revealed that both NEU and BAS counts significantly mediated the association between PAH mixtures and LFHL.

Our study indicated that mixed PAH exposure was positively associated with hearing loss. Few previous epidemiological studies have previously investigated the relationship of PAHs with hearing loss (18, 19). For example, Chou et al. reported a positive association between urinary PAH metabolites and hearing thresholds in adults using a multivariate linear regression model (19). Similarly, Li et al. found positive associations of 2-OHFLU, 3-OHFLU and 2 & 3-OHPHE with hearing loss in U.S. adults using logistic regression models (18). However, these studies did not consider the combined effects of PAHs on hearing loss or potential collinearity between metabolites (40, 45). In contrast, this study used WQS and BKMR models to address the limitations. These models are novel approaches for evaluating the relationship between co-exposure to multiple, potentially correlated pollutants and health outcomes. Both WQS and BKMR models can reduce dimensionality and mitigate multicollinearity (40). Notably, aside from the linear relationship observed for LFHL, ∑OH-PAHs were found to be non-linearly associated with HL and HFHL. Specifically, when the urinary ∑OH-PAHs concentration exceeded thresholds of 4.477, 4.581, and 4.842 ng/g Cr, the odds of LFHL, HL, and HFHL significantly increased, respectively. These findings suggested that PAH mixed exposure above cut-off levels may be harmful to the general population. Additionally, the BKMR model can better capture these non-linear and non-additive relationships between exposure and response, which also elucidates interactions among exposures (38). A more thorough understanding of the relationship between PAH mixtures and hearing loss in real-world scenarios can be obtained by using these advanced statistical models simultaneously.

Previous studies have identified naphthalene as a primary source of PAHs for U.S. adults (46, 47). Similar results were observed in our study. 1-OHNAP and 2-OHNAP are two positional isomers of naphthol, and 1-OHNAP is the primary metabolite of naphthalene (47). According to the WQS index model, 1-OHNAP was the primary contributor to the association between PAH mixtures and hearing loss. Naphthalene mainly originates from sources such as insecticides, herbicides, vehicle emissions, camphor mothballs, biomass fuels or wood combustion, and tobacco smoke (48). Therefore, the positive association between 1-OHNAP levels and hearing loss may be linked to the greater likelihood of non-occupational exposure to naphthalene in adults' daily lives. In contrast, 1-OHPYR was negatively associated with hearing loss in BKMR, which was consistent with the results of the multivariable regression model. This may be due to 1-OHPYR being the only high-molecular-weight PAH metabolite in urine, and its metabolic pathway may differ from other low-molecular-weight substances (30, 49). In the metabolism of PAHs, larger molecular weight PAHs (such as OHPYR) are predominantly excreted via feces, while smaller PAHs (containing 2-4 aromatic rings) are excreted through urine (50). Therefore, low-level exposure to OHPYR may not be detectable via urinary biomonitoring.

The common mechanism of hearing loss is cochlear degeneration, characterized by damage to the inner ear hair cells in the Corti organ (1). This damage is often attributed to oxidative stress, chronic inflammation, vasoconstriction, and other factors (51, 52). Although the mechanism of PAH exposure and hearing loss has not been fully elucidated, several potential pathways may be proposed. First, oxidative stress caused by PAHs (53) may lead to hair cell apoptosis (54), hinder neural signal transmission, and ultimately contribute to hearing loss (52). Second, the effects of PAHs on hearing function may be related to the elevation of blood lipid levels (55), which could narrow blood vessels, increase blood viscosity and cause cell loss in the spiral ligament (SL) and spiral ganglion (SG) of the cochlear basal (56). Third, PAHs and their metabolites may inhibit the activity of thyroid hormone receptors (57). Relevant evidence suggests that developmental hypothyroidism is positively associated with hearing dysfunction in rats (58).

Leukocytes were regarded as prognostic indicators of inflammation in the inner ear, as they are easier to detect and more cost-effective (59). Previous studies have reported positive association between PAH mixtures and neutrophil counts (25), as well as elevated leukocyte counts increased the hearing thresholds (60). However, the mediating role of inflammatory cells in the relationship between PAHs and hearing loss remains unproven. In the present study, we found that both NEU and BAS significantly mediated the association between PAH mixtures and LFHL, suggesting the mediating role of inflammatory response in PAH-related hearing loss. Several pathological pathways may contribute to inflammation related hearing loss (61). NEU and BAS could increase the release of proinflammatory cytokines, including TNF-α, IL-4, and IL-8 (62, 63). These natural inflammatory cytokines broke down the tight junctions in the vessel, opened the blood-labyrinth barrier, and caused hearing loss (64). It was also reported that inflammatory cells contributed to form atherosclerosis in cochlea, which induced hair cell hypoxia, further caused hearing loss (59, 65).

This study has several strengths. To our knowledge, this is the first study to investigate the combined effects of PAHs on hearing loss in U.S. adults. Additionally, three statistical methods were employed, and the consistency of results across these models highlights the robustness of the findings. Simultaneous use of these models can mitigate multicollinearity and uncover the non-linear and non-additive associations between exposures and outcomes (38). Furthermore, to clarify the underlying mechanism, mediation analysis was conducted to demonstrate the mediating role of inflammation in the relationship between PAH mixtures and hearing loss. However, there are several limitations to consider. First, no causal inference can be drawn from the data as they were cross-sectional. Second, excluding individuals who did not meet the study's inclusion criteria may limit the representativeness of the population and the generalizability of results. So, inverse probability weighted analysis was applied to minimize the potential selection bias. Third, noise exposure definition in our study was based on self-reports, and there was a lack of objective methods to measure noise exposure. Fourth, the lack of genetic data in the NHANES database prevented us from controlling genetic factors related to hearing. Finally, as the study was based on the data from the U.S. nationally representative survey, caution should be exercised when generalizing the findings to other populations, such as regions with different levels of PAH exposure. Therefore, further cohort studies are desired to validate the relationship between PAH mixtures and hearing loss.

## 5 Conclusions

We provide evidence that mixed PAH exposure is positively associated with hearing loss through the use of multiple models. 1-OHNAP is identified as the primary contributor to the positive association between PAH mixtures and hearing loss. Additionally, inflammatory cell markers significantly mediate the association between PAH mixtures and LFHL. Our study provides valuable insight into the underlying mechanisms, and further studies are needed to validate our findings.

## Data Availability

The datasets presented in this study can be found in online repositories. The names of the repository/repositories and accession number(s) can be found below: https://www.cdc.gov/nchs/nhanes/index.htm.
