# Supplementary material for: Association of mixed polycyclic aromatic hydrocarbons exposure with hearing loss and the mediating role of blood cell markers of inflammation in U.S. adults
Source: Front Public Health. 2024 Nov 27;12:1410601. doi: 10.3389/fpubh.2024.1410601 (PMC11631931; doi:10.3389/fpubh.2024.1410601)
Supplement: Supplementary file 1 [file Data_Sheet_1.docx]

**Contents**

**Fig. S1** Flowchart of participant selection. NHANES (2001-2002, 2003-2004 and 2011-2012).

**Table S1** Distribution of polycyclic aromatic hydrocarbons exposures (N = 1409), NHANES (2001-2002, 2003-2004 and 2011-2012).

**Fig. S2** The dose–response association between ΣOH-PAHs and hearing loss in restricted cubic spline models.

**Table S2** Weighted quantiles sum (WQS) regression index weights estimated in model (N=1,409), NHANES (2001-2002, 2003-2004 and 2011-2012)

**Fig. S3** Bivariate exposure response functions of every two exposures in HL(A), HFHL(B) and, LFHL(C) in BKMR models.

**Table S3** Association between inflammatory blood cell markers and hearing loss (N = 1409), NHANES (2001-2002, 2003-2004 and 2011-2012).

**Fig. S4** Linear regression results between log-transformed OH-PAHs and inflammatory blood cell markers.

**Fig. S5** Mediation analysis of blood cell markers of inflammation on the association between PAH mixtures and HL.

**Fig. S6** Mediation analysis of blood cell markers of inflammation on the association between PAH mixtures and HFHL.

**Table S4** Association between OH-PAHs exposure and hearing loss (N = 1409), NHANES (2001-2002, 2003-2004 and 2011-2012)

**Table S5** Association between the WQS index and hearing loss, NHANES (2002-2004 and 2011-2012).

**Fig. S7** The combined effects of OH-PAH mixtures on HL (A), HFHL (B), and LFHL (C) in BKMR models, NHANES (2003-2004 and 2011-2012)

**Fig. S8** The univariate exposure‒response functions and 95% confidence intervals for each OH-PAH with HL (A), HFHL (B) and LFHL (C) when fixing other chemicals at their 50th percentile, NHANES (2003-2004 and 2011-2012)

**Fig. S9** Bivariate exposure response functions of every two exposures in HL(A), HFHL(B) and, LFHL(C) in BKMR models, NHANES (2003-2004 and 2011-2012)


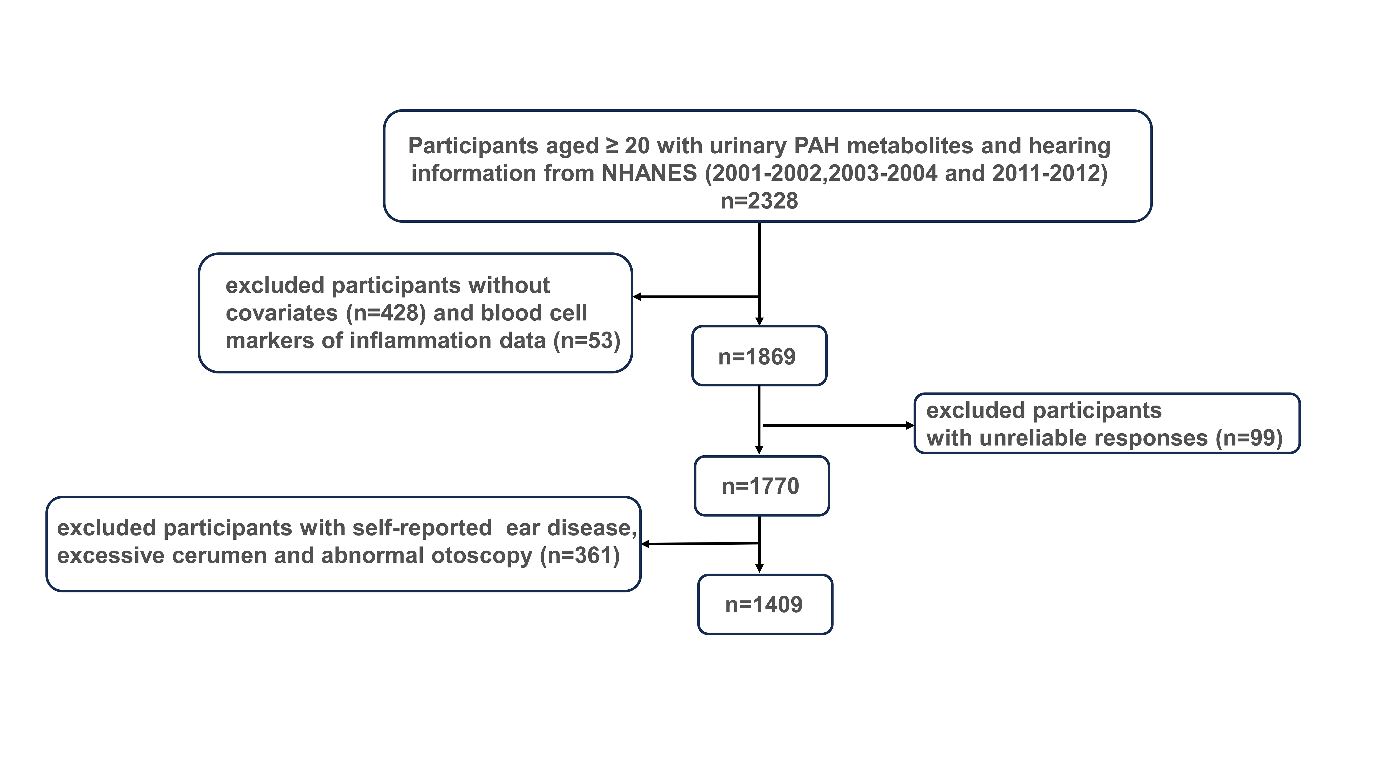


**Fig. S1** Flowchart of participant selection. NHANES (2001-2002, 2003-2004 and 2011-2012). HFHL indicates high-frequency hearing loss; HL, hearing loss; LFHL, low-frequency hearing loss

| **Table S1** Distribution of polycyclic aromatic hydrocarbons exposures (N = 1,409), NHANES (2001-2002, 2003-2004 and 2011-2012). | | | | | | | | |
| --- | --- | --- | --- | --- | --- | --- | --- | --- |
| Urinary PAH Metabolites, ng/L | Detection Frequency | GM | Mean | Percentile | | | | |
|  |  |  |  | 5th | 25th | 50th | 75th | 95th |
| 1-OHNAP | 93.8% | 2121.3 | 17370.3 | 238.5 | 718.0 | 1741.0 | 5679.5 | 25617.5 |
| 2-OHNAP | 94.2% | 3881.9 | 7990.3 | 489.0 | 1556.5 | 3892.0 | 10005.5 | 28542.5 |
| 3-OHFLU | 93.9% | 117.0 | 339.8 | 15.0 | 43.8 | 95.0 | 286.0 | 1628.0 |
| 2-OHFLU | 94.1% | 291.4 | 658.8 | 43.0 | 125.0 | 257.0 | 645.0 | 2692.5 |
| 1-OHPHE | 93.9% | 136.8 | 224.2 | 27.0 | 73.0 | 135.2 | 265.0 | 627.2 |
| 1-OHPYR | 94.0% | 88.9 | 187.3 | 12.0 | 41.0 | 87.0 | 184.0 | 597.0 |
| 2 & 3-OHPHE | 93.2% | 155.7 | 298.6 | 28.0 | 77.0 | 149.0 | 311.0 | 876.5 |
| 1-OHNAP, 1-hydroxynaphthalene; 1-OHPHE, 1-hydroxyphenanthrene; 1-OHPYR, 1-hydroxypyrene; 2 & 3-OHPHE, 2 & 3-hydroxyphenanthrene; 2-OHFLU, 2-hydroxyfluorene; 2-OHNAP, 2-hydroxynaphthalene; 3-OHFLU, 3-hydroxyfluorene; GM: geometric mean, PAH, polycyclic aromatic hydrocarbon | | | | | | | | |


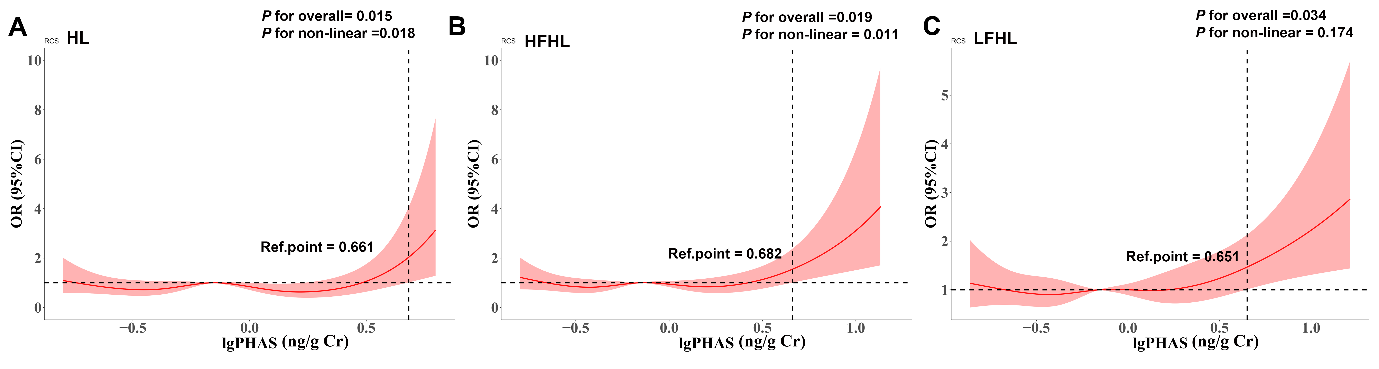


**Fig. S2** The dose–response association between log-transformed ΣOH-PAHs and hearing loss in restricted cubic spline models. A, HL. B, HFHL. C, LFHL. The estimated ORs are indicated by solid lines and 95% CIs by shaded areas. All models were adjusted for age, sex, race/ethnicity, education level, marital status, PIR, BMI, drinking, smoking, diabetes, hypertension, cardiovascular disease, occupational, firearm, recreational noise, and use of ototoxic medication.

| **Table S2** Weighted quantiles sum (WQS) regression index weights estimated in model (N=1,409), NHANES (2001-2002, 2003-2004 and 2011-2012) |
| --- |

| **Urinary PAH Metabolites,**  **ng/g Cr** | **Estimated Weights for WQS Index** | | |
| --- | --- | --- | --- |
|  | **HL** | **HFHL** | **LFHL** |
| 1-OHPHE | 0.106 | 0.004 | 0.068 |
| 2-OHNAP | 0.148 | 0.048 | 0.027 |
| 2 & 3-OHPHE | 0.080 | 0.097 | 0.074 |
| 3-OHFLU | 0.246 | 0.582 | 0.258 |
| 1-OHNAP | **0.287** | 0.072 | **0.429** |
| 2-OHFLU | 0.123 | **0.646** | 0.133 |
| 1-OHPYR | 0.010 | 0.057 | 0.010 |

All models were adjusted for age, sex, race/ethnicity, education level, marital status, PIR, BMI, drinking, smoking, diabetes, hypertension, cardiovascular disease, occupational, firearm, recreational noise, and use of ototoxic medication

1-OHNAP, 1-hydroxynaphthalene; 1-OHPHE, 1-hydroxyphenanthrene; 1-OHPYR, 1-hydroxypyrene; 2 & 3-OHPHE, 2 & 3-hydroxyphenanthrene; 2-OHFLU, 2-hydroxyfluorene; 2-OHNAP, 2-hydroxynaphthalene; 3-OHFLU, 3-hydroxyfluorene; HFHL, high-frequency hearing loss; HL, hearing loss; LFHL, low-frequency hearing loss


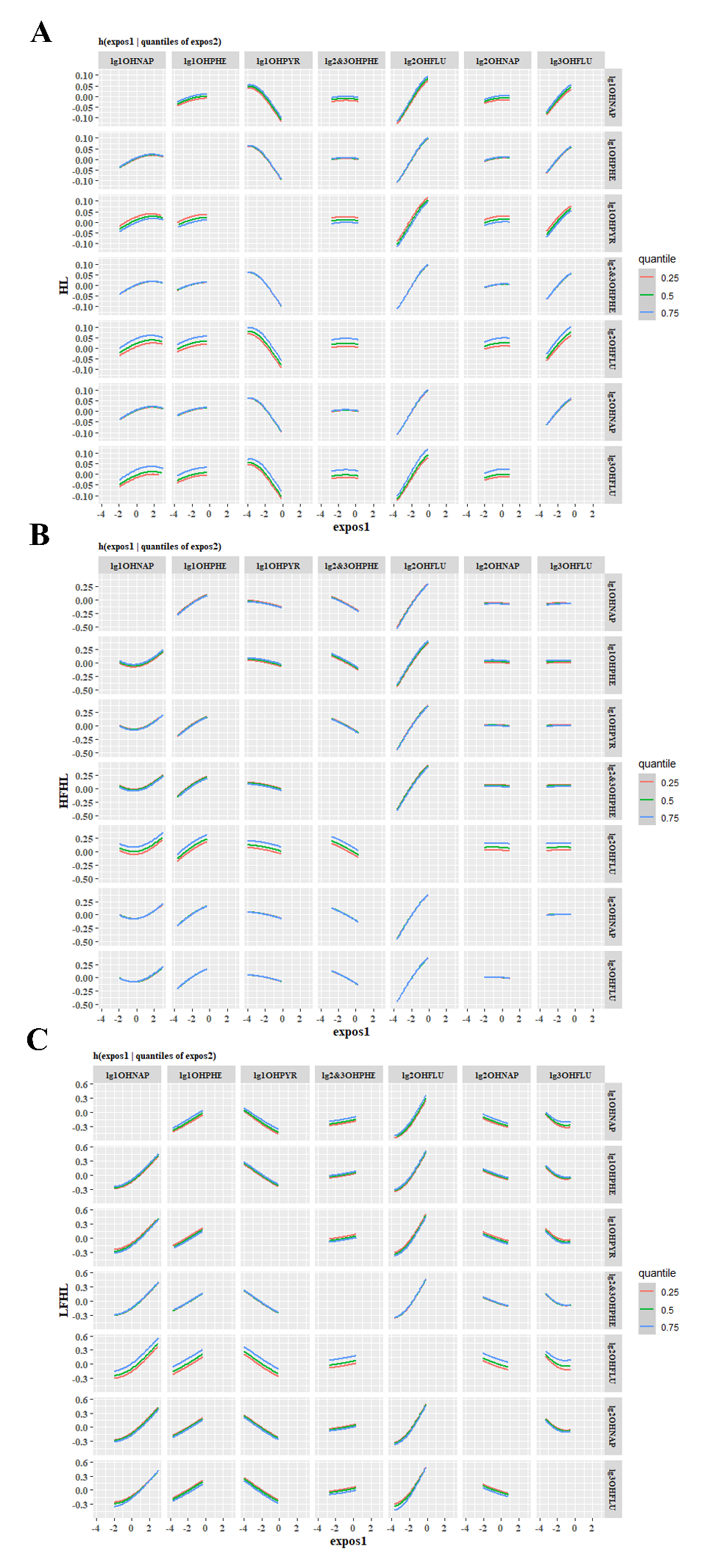


**Fig. S3** Bivariate exposure response functions of every two exposures in HL(A), HFHL(B) and, LFHL(C) in BKMR models. Figures show a relationship of individual OH-PAH with HL(A), HFHL(B) and, LFHL(C), when an individual OH-PAH exposure was at its 75th percentile as compared to its 25th percentile, and the other OH-PAHs were fixed at a specific exposure percentile (25th, 50th, or 75th, respectively). All models were adjusted for age, sex, race/ethnicity, education level, marital status, PIR, BMI, drinking, smoking, diabetes, hypertension, cardiovascular disease, occupational, firearm, recreational noise, and use of ototoxic medication. 1-OHNAP indicates 1-hydroxynaphthalene; 1-OHPHE, 1-hydroxyphenanthrene; 1-OHPYR, 1-hydroxypyrene; 2 & 3-OHPHE, 2 & 3-hydroxyphenanthrene; 2-OHFLU, 2-hydroxyfluorene; 2-OHNAP, 2-hydroxynaphthalene; 3-OHFLU, 3-hydroxyfluorene; HFHL, high-frequency hearing loss; HL, hearing loss; LFHL, low-frequency hearing loss


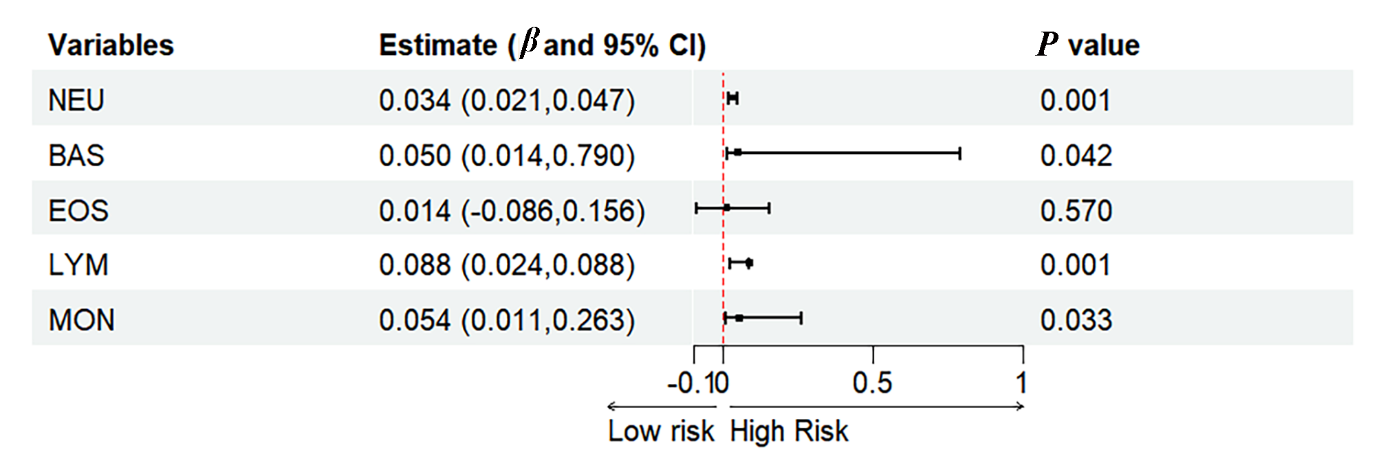


**Fig. S4** Linear regression results between log transformed ΣOH-PAHs and inflammatory blood cell markers. The models were adjusted for age, sex, race/ethnicity, education level, marital status, PIR, BMI, drinking, smoking, diabetes, hypertension, cardiovascular disease, occupational, firearm, recreational noise, and using of ototoxic medication. BAS indicates basophil count; EOS, eosinophilic count; LYM, lymphocyte count; MON, monocyte count; NEU, neutrophil count

**Table S3** Association between inflammatory blood cell markers and hearing loss (N = 1409), NHANES (2001-2002, 2003-2004 and 2011-2012).

| **Inflammatory blood cell markers, 1000 cells/μl** | **HL** | |  | **HFHL** | |  | **LFHL** | |  |
| --- | --- | --- | --- | --- | --- | --- | --- | --- | --- |
|  | **OR (95% CI)** | ***p*** |  | **OR (95% CI)** | ***p*** |  | **OR (95% CI)** | ***p*** |  |
| **NEU** | **1.090 (1.011-1.176)** | **0.026^*^** |  | 1.015 (0.929-1.109) | 0.736 |  | **1.125 (1.048 -1.208)** | **0.001^*^** |  |
| **BAS** | **8.324 (1.103-62.813)** | **0.040^*^** |  | 0.796 (0.073-7.438) | 0.737 |  | **19.927 (3.030-131.639)** | **0.002^*^** |  |
| **EOS** | 1.376 (0.706-2.684) | 0.348 |  | 1.151 (0.530-2.499) | 0.722 |  | 1.639 (0.878-3.059) | 0.121 |  |
| **LYM** | 0.935 (0.774-1.130) | 0.488 |  | 0.948 (0.756-1.189) | 0.644 |  | 0.942 (0.792-1.121) | 0.501 |  |
| **MON** | 1.105 (0.541-2.261) | 0.784 |  | 0.947 (0.398-2.253) | 0.903 |  | 1.536 (0.786-3.003) | 0.209 |  |

The model was adjusted for age, sex, race/ethnicity, education level, marital status, PIR, BMI, drinking, smoking, diabetes, hypertension, cardiovascular disease, occupational, firearm, recreational noise, and use of ototoxic medication. BAS indicates basophil count; EOS, eosinophilic count; LYM, lymphocyte count; MON, monocyte count; NEU, neutrophil count; HFHL, high-frequency hearing loss; HL, hearing loss; LFHL, low-frequency hearing loss; *OR*: odds ratios

**p* < 0.05, ***p* < 0.01


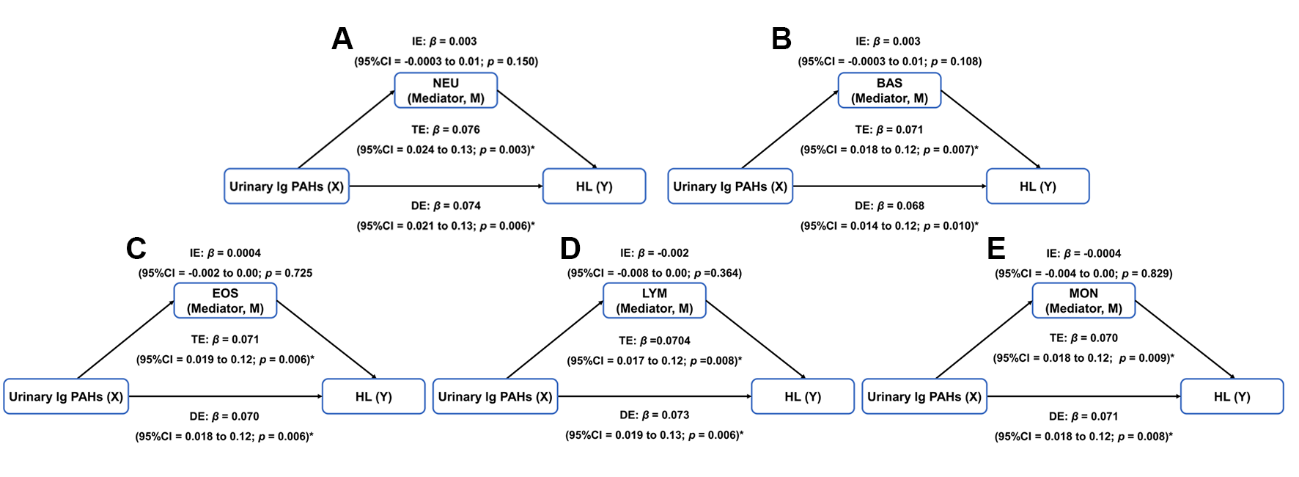


**Fig. S5** Mediation analysis of blood cell markers of inflammation on the association between PAH mixtures and HL. A, NEU. B, BAS. C, EOS. D, LYM. E, MON. BAS indicates basophil count; DE, effect; EOS, eosinophilic count; HL, hearing loss; IE, indirect effect; LYM, lymphocyte count; MON, monocyte count; NEU, neutrophil count; PAH, polycyclic aromatic hydrocarbon; TE, total effect. * *p* < 0.05


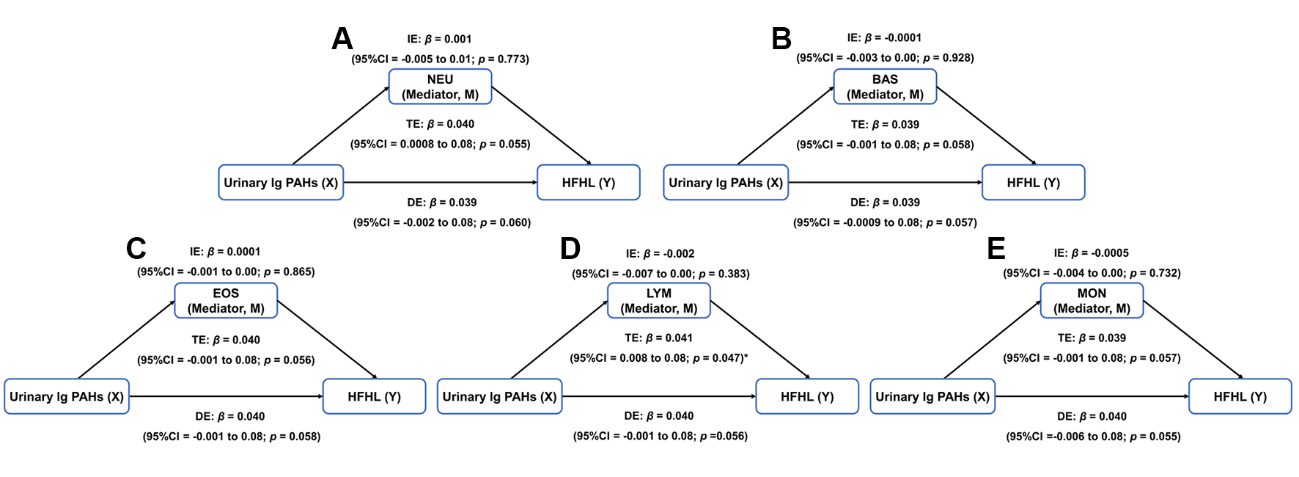


**Fig. S6** Mediation analysis of blood cell markers of inflammation on the association between PAH mixtures and HFHL. A, NEU. B, BAS. C, EOS. D, LYM. E, MON. BAS indicates basophil count; DE, effect; EOS, eosinophilic count; HFHL, high-frequency hearing loss; IE, indirect effect; LYM, lymphocyte count; MON, monocyte count; NEU, neutrophil count; PAH, polycyclic aromatic hydrocarbon; TE, total effect. * *p* < 0.05

**Table S4** Association between OH-PAHs exposure and hearing loss (N = 1409), NHANES (2001-2002, 2003-2004 and 2011-2012).

| **Urinary PAH Metabolites, ng/g Cr** | **HL** | |  | **HFHL** | |  | **LFHL** | |  |
| --- | --- | --- | --- | --- | --- | --- | --- | --- | --- |
|  | **OR (95% CI)** | ***p*** |  | **OR (95% CI)** | ***p*** |  | **OR (95% CI)** | ***p*** |  |
| **1-OHPYR** |  | |  |  |  |  |  |  |  |
| Continuous log-transformed | **0.508 (0.324-0.797)** | **0.003^*^** |  | 0.788 (0.456-1.360) | 0.392 |  | **0.628 (0.417-0.946)** | **0.026^*^** |  |
| Quartile1 | ref | |  | ref | |  | ref | |  |
| Quartile2 | **0.655 (0.451-0.950)** | **0.026^*^** |  | 1.077 (0.678-1.709) | 0.754 |  | 0.841 (0.591-1.197) | 0.337 |  |
| Quartile3 | 0.706 (0.470-1.067) | 0.099 |  | 0.856 (0.514-1.426) | 0.551 |  | 0.885 (0.598-1.312) | 0.544 |  |
| Quartile4 | **0.485 (0.296-0.795)** | **0.004**^*^ |  | 0.756 (0.407-1.404) | 0.375 |  | 0.784 (0.486-1.265) | 0.318 |  |
| *P* for trend | **0.020^*^** | |  | 0.804 | |  | 0.499 | |  |
| **1-OHNAP** |  |  |  |  |  |  |  |  |  |
| Continuous log-transformed | 1.068 (0.796-1.436) | 0. 658 |  | 1.028 (0.725-1.458) | 0.467 |  | 1.259 (0.959-1.652) | 0.097 |  |
| Quartile1 | ref | |  | ref | |  | ref | |  |
| Quartile2 | 1.060 (0.735-1.529) | 0.756 |  | 0.772 (0.466-1.309) | 0.217 |  | 1.224 (0.777-1.929) | 0.362 |  |
| Quartile3 | 0.886 (0.600-1.308) | 0.542 |  | **0.600 (0.383-0.941)** | **0.026^*^** |  | 1.050 (0.635-1.734) | 0.462 |  |
| Quartile4 | 1.133 (0.683-1.879) | 0.628 |  | 1.030 (0.550-1.928) | 0.927 |  | 1.348 (0.757-2.400) | 0.101 |  |
| *P* for trend | 0.692 | |  | 0.467 | |  | 0.125 | |  |
| **2-OHNAP** |  |  |  |  |  |  |  |  |  |
| Continuous log-transformed | 1.139 (0.775-1.674) | 0.508 |  | 1.104 (0.706-1.727) | 0.663 |  | 0.953 (0.666-1.364) | 0.793 |  |
| Quartile1 | ref | |  | ref | |  | ref | |  |
| Quartile2 | 0.941 (0.659-1.343) | 0.736 |  | 1.079 (0.707-1.731) | 0.724 |  | 0.972 (0.691-1.366) | 0.868 |  |
| Quartile3 | 1.195 (0.808-1.766) | 0.372 |  | 1.094 (0.687-1.429) | 0.705 |  | 1.039 (0.720-1.500) | 0.839 |  |
| Quartile4 | 1.037 (0.642-1.674) | 0.881 |  | 1.051 (0.612-1.806) | 0.856 |  | 0.831 (0.536-1.288) | 0.408 |  |
| *P* for trend | 0.655 | |  | 0.674 | |  | 0.419 | |  |
| **1-OHPHE** |  |  |  |  |  |  |  |  |  |
| Continuous log-transformed | 1.766 (0.918-3.396) | 0.088 |  | 1.841 (0.788-4.304) | 0.159 |  | 1.564 (0.835-2.930) | 0.162 |  |
| Quartile1 | ref | |  | ref | |  | ref | |  |
| Quartile2 | 1.418 (0.976-2.060) | 0.067 |  | 1.363 (0.880-2.111) | 0.164 |  | 1.302 (0.898-1.889) | 0.163 |  |
| Quartile3 | 1.411 (0.911-2.185) | 0.123 |  | 1.690 (0.997-2.864) | 0.051 |  | 1.312 (0.869-1.979) | 0.196 |  |
| Quartile4 | **1.812 (1.041-3.152)** | **0.036*** |  | 1.228 (0.644-2.342) | 0.532 |  | 1.441 (0.860-2.416) | 0.165 |  |
| *P* for trend | 0.084 | |  | 0.308 | |  | 0.429 | |  |
| **2 & 3-OHPHE** |  |  |  |  |  |  |  |  |  |
| Continuous log-transformed | 0.731 (0.409-1.305) | 0.324 |  | 0.611 (0.284-1.315) | 0.207 |  | 0.991 (0.597 -1.643) | 0.971 |  |
| Quartile1 | ref | |  | ref | |  | ref | |  |
| Quartile2 | 0.935 (0.633-1.382) | 0.737 |  | 1.155 (0.726-1.836) | 0.542 |  | 0.808 (0.552-1.183) 0.273 | 0.273 |  |
| Quartile3 | 0.932 (0.578-1.502) | 0.771 |  | 1.367 (0.766-2.438) | 0.290 |  | 0.818 (0.521-1.286) | 0.384 |  |
| Quartile4 | 0.853 (0.470-1.548) | 0.600 |  | 1.273 (0.609-2.661) | 0.520 |  | 1.040 (0.603-1.792) | 0.890 |  |
| *P* for trend | 0.289 | |  | 0.724 | |  | 0.897 | |  |
| **3-OHFLU** |  |  |  |  |  |  |  |  |  |
| Continuous log-transformed | 0.869 (0.437-1.727) | 0.689 |  | 0.861 (0.385-1.925) | 0.715 |  | 0.644 (0.346-1.201) | 0.166 |  |
| Quartile1 | ref | |  | ref | |  | ref | |  |
| Quartile2 | 1.017 (0.677-1.529) | 0.935 |  | 1.027 (0.629-1.677) | 0.916 |  | 0.992 (0.672-1.464) | 0.969 |  |
| Quartile3 | 0.979 (0.575-1.665) | 0.937 |  | 0.733 (0.382-1.404) | 0.348 |  | 1.063 (0.649-1.744) | 0.807 |  |
| Quartile4 | 1.050 (0.476-2.320) | 0.903 |  | 0.790 (0.286-2.182) | 0.650 |  | 0.869 (0.410-1.839) | 0.711 |  |
| *P* for trend | 0.828 | |  | 0.712 | |  | 0.756 | |  |
| **2-OHFLU** |  |  |  |  |  |  |  |  |  |
| Continuous log-transformed | **2.500 (1.088-5.744)** | **0.031^*^** |  | 2.070 (0.811-5.286) | 0.128 |  | **2.521 (1.164-5.456)** | **0.019^*^** |  |
| Quartile1 | ref | |  | ref | |  | ref | |  |
| Quartile2 | 0.980 (0.646-1.488) | 0.924 |  | 1.126 (0.691-1.834) | 0.634 |  | 0.929 (0.620-1.393) | 0.722 |  |
| Quartile3 | 1.075 (0.616-1.876) | 0.798 |  | 1.755 (0.909-3.387) | 0.094 |  | 0.793 (0.463-1.360) | 0.400 |  |
| Quartile4 | 1.871 (0.861-4.066) | 0.114 |  | 1.727 (0.659-4.527) | 0.266 |  | 1.640 (0.774-3.470) | 0.196 |  |
| *P* for trend | 0.089 | |  | 0.409 | |  | 0.137 | |  |
| **ΣOH-PAHs** |  |  |  |  |  |  |  |  |  |
| Continuous log-transformed | **1.480 (1.092-2.004)** | **0.011^*^** |  | **1.524 (1.049-2.215)** | **0.027^*^** |  | **1.495 (1.135 -1.968)**  **..** | **0.004^*^** |  |
| Quartile1 | ref | |  | ref | |  | ref | |  |
| Quartile2 | **1.644 (1.167-2.316)** | **0.004^*^** |  | **1.767 (1.179-2.651)** | **0.006^*^** |  | **1.420 (0.020-1.978)** | **0.038^*^** |  |
| Quartile3 | 1.249 (0.882-1.767) | 0.210 |  | 1.197 (0.804-1.782) | 0.376 |  | 1.215 (0.874 1.689) | 0.246 |  |
| Quartile4 | **1.787 (1.232-2.594)** | **0.002^*^** |  | 1.536 (0.986-2.394) | 0.058 |  | **1.837 (1.304-2.589)** | **0.001^*^** |  |
| *P* for trend | **0.016^*^** | |  | 0.462 | |  | **0.001^*^** | |  |

The model was adjusted for age, sex, race/ethnicity, education level, marital status, PIR, BMI, drinking, smoking, diabetes, hypertension, cardiovascular disease, occupational, firearm, recreational noise, and use of ototoxic medication.1-OHNAP indicates 1-hydroxynaphthalene; 1-OHPHE, 1-hydroxyphenanthrene; 1-OHPYR, 1-hydroxypyrene; 2 & 3-OHPHE, 2 & 3-hydroxyphenanthrene; 2-OHFLU, 2-hydroxyfluorene; 2-OHNAP, 2-hydroxynaphthalene; 3-OHFLU, 3-hydroxyfluorene; ∑OH-PAHs, total of PAH metabolites; *CI*, confidence interval; HFHL, high-frequency hearing loss; HL, hearing loss; LFHL, low-frequency hearing loss; *OR*: odds ratios; PAHs, polycyclic aromatic hydrocarbon

*P* for trend across quartiles of urinary PAH metabolites was tested by including the median of each quartile of urinary PAH metabolites as a continuous variable in models. **p* < 0.05, ***p* < 0.01

**Table S5** Association between the WQS index and hearing loss, NHANES (2003-2004 and 2011-2012).

|  | OR | 95% CI of OR | *p* |
| --- | --- | --- | --- |
| HL | 1.445 | 1.062-1.967 | **0.019^*^** |
| HFHL | 1.282 | 0.854-1.924 | **0.231** |
| LFHL | 1.776 | 1.000-3.154 | **0.049^*^** |

The WQS model was adjusted for age, sex, race/ethnicity, education level, marital status, PIR, BMI, drinking, smoking, diabetes, hypertension, cardiovascular disease, occupational, firearm, recreational noise, and use of ototoxic medication. OR estimates represent the odds ratios of hearing loss when the WQS index of PAH mixtures was increased by 1 tertile

CI indicates confidence interval; HFHL, high-frequency hearing; HL, hearing loss; LFHL, low-frequency hearing loss; OR, odds ratios

**p* < 0.05


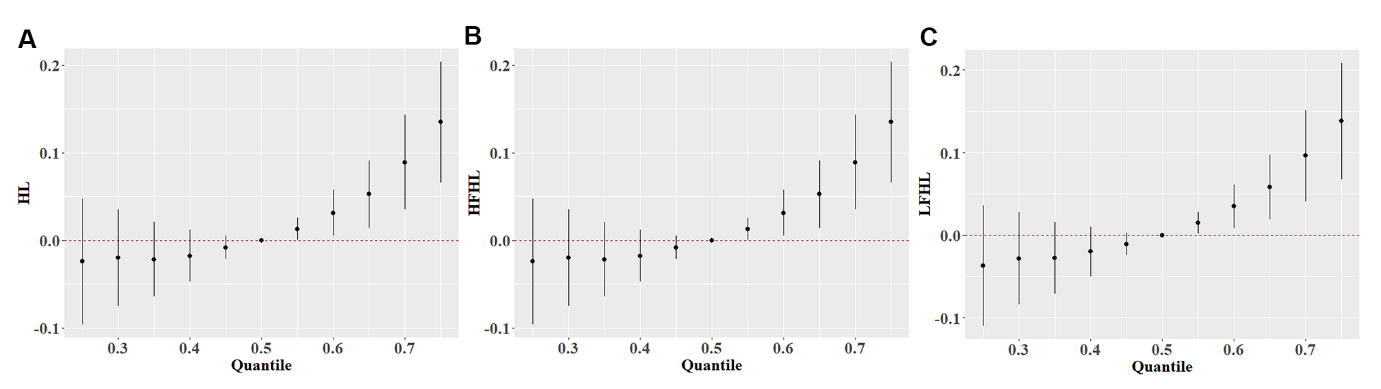


**Fig. S7** The combined effects of OH-PAH mixtures on HL (A), HFHL (B), and LFHL (C) in BKMR models, NHANES (2003-2004 and 2011-2012). All models were adjusted age, sex, race/ethnicity, education level, marital status, PIR, BMI, drinking, smoking, diabetes, hypertension, cardiovascular disease, occupational, firearm, recreational noise, and use of ototoxic medication. HFHL indicates high-frequency hearing loss; HL, hearing loss; LFHL, low-frequency hearing loss


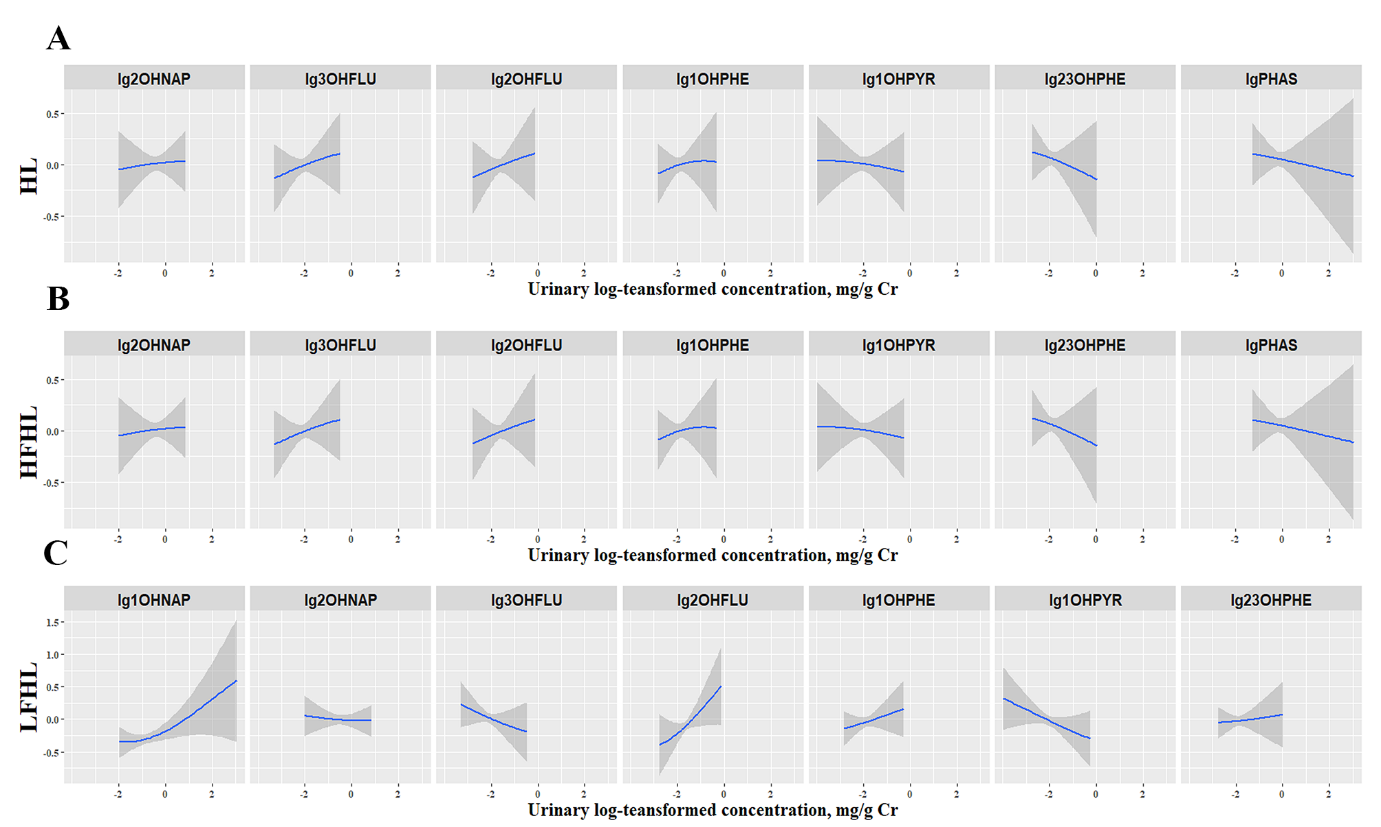


**Fig. S8** The univariate exposure‒response functions and 95% confidence intervals for each OH-PAH with HL (A), HFHL (B) and LFHL (C) when fixing other chemicals at their 50th percentile, NHANES (2003-2004 and 2011-2012). The shaded areas represent 95% confidence intervals. All models were adjusted for age, sex, race/ethnicity, education level, marital status, PIR, BMI, drinking, smoking, diabetes, hypertension, cardiovascular disease, occupational, firearm, recreational noise, and use of ototoxic medication. 1-OHNAP indicates 1-hydroxynaphthalene; 1-OHPHE, 1-hydroxyphenanthrene; 1-OHPYR, 1-hydroxypyrene; 2 & 3-OHPHE, 2 & 3-hydroxyphenanthrene; 2-OHFLU, 2-hydroxyfluorene; 2-OHNAP, 2-hydroxynaphthalene; 3-OHFLU, 3-hydroxyfluorene; HFHL, high-frequency hearing loss; HL, hearing loss; LFHL, low-frequency hearing loss


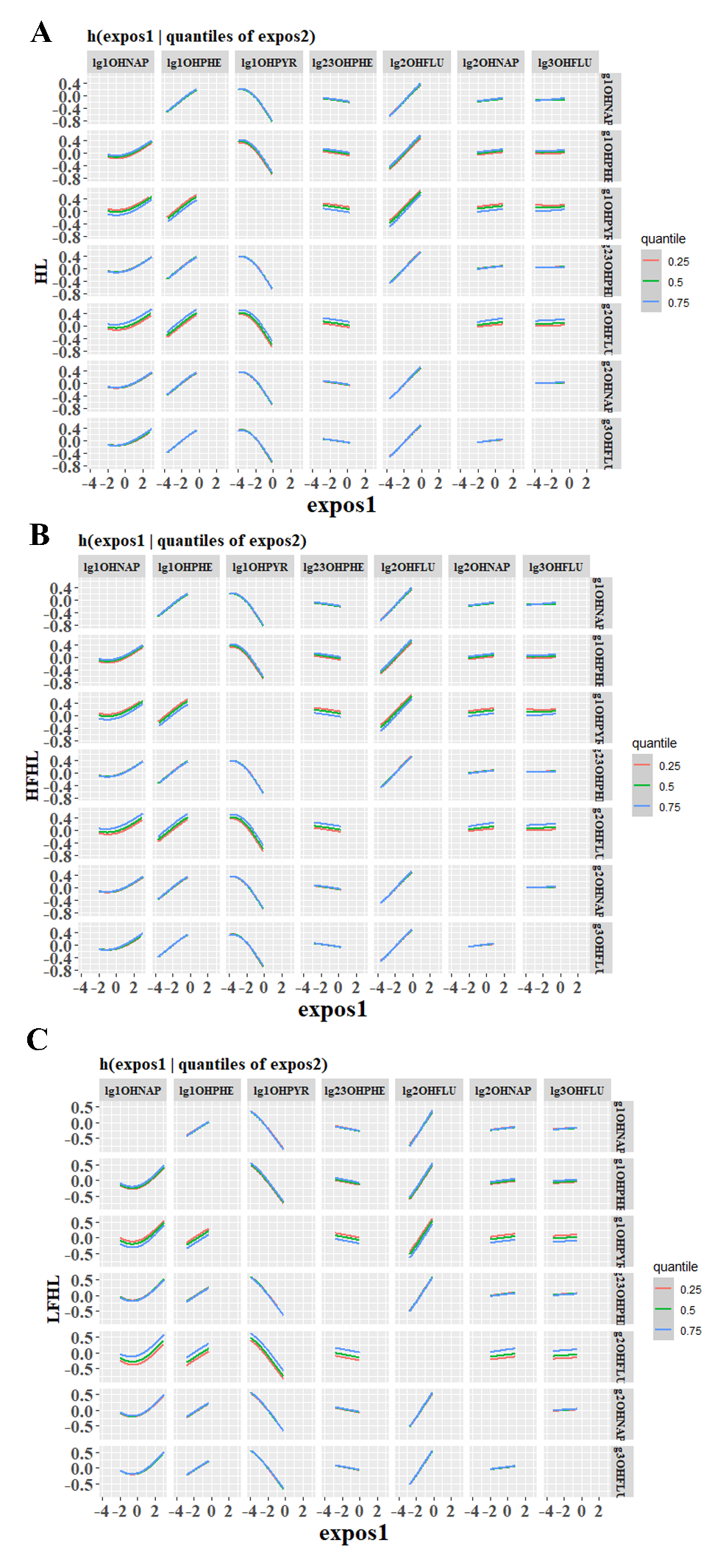


**Fig. S9** Bivariate exposure response functions of every two exposures in HL(A), HFHL(B) and, LFHL(C) in BKMR models, NHANES (2003-2004 and 2011-2012). Figures show a relationship of individual OH-PAH with HL(A), HFHL(B) and, LFHL(C), when an individual OH-PAH exposure was at its 75th percentile as compared to its 25th percentile, and the other OH-PAHs were fixed at a specific exposure percentile (25th, 50th, or 75th, respectively). All models were adjusted for age, sex, race/ethnicity, education level, marital status, PIR, BMI, drinking, smoking, diabetes, hypertension, cardiovascular disease, occupational, firearm, recreational noise, and use of ototoxic medication. 1-OHNAP indicates 1-hydroxynaphthalene; 1-OHPHE, 1-hydroxyphenanthrene; 1-OHPYR, 1-hydroxypyrene; 2 & 3-OHPHE, 2 & 3-hydroxyphenanthrene; 2-OHFLU, 2-hydroxyfluorene; 2-OHNAP, 2-hydroxynaphthalene; 3-OHFLU, 3-hydroxyfluorene; HFHL, high-frequency hearing loss; HL, hearing loss; LFHL, low-frequency hearing loss
